# Framework for evaluating photon-counting detectors under pile-up conditions

**DOI:** 10.1117/1.JMI.11.S1.S12802

**Published:** 2024-05-24

**Authors:** David Leibold, Stefan J. van der Sar, Marlies C. Goorden, Dennis R. Schaart

**Affiliations:** a Delft University of Technology, Department of Radiation Science and Technology, Delft, The Netherlands; b HollandPTC, Delft, The Netherlands

**Keywords:** photon-counting X-ray detectors, non-linear systems, pile-up, point spread function, small-signal analysis, medical imaging

## Abstract

**Purpose:**

While X-ray photon-counting detectors (PCDs) promise to revolutionize medical imaging, theoretical frameworks to evaluate them are commonly limited to incident fluence rates sufficiently low that the detector response can be considered linear. However, typical clinical operating conditions lead to a significant level of pile-up, invalidating this assumption of a linear response. Here, we present a framework that aims to evaluate PCDs, taking into account their non-linear behavior.

**Approach:**

We employ small-signal analysis to study the behavior of PCDs under pile-up conditions. The response is approximated as linear around a given operating point, determined by the incident spectrum and fluence rate. The detector response is subsequently described by the proposed perturbation point spread function (pPSF). We demonstrate this approach using Monte-Carlo simulations of idealized direct- and indirect-conversion PCDs.

**Results:**

The pPSFs of two PCDs are calculated. It is then shown how the pPSF allows to determine the sensitivity of the detector signal to an arbitrary lesion. This example illustrates the detrimental influence of pile-up, which may cause non-intuitive effects such as contrast/contrast-to-noise ratio inversion or cancellation between/within energy bins.

**Conclusions:**

The proposed framework permits quantifying the spectral and spatial performance of PCDs under clinically realistic conditions at a given operating point. The presented example illustrates why PCDs should not be analyzed assuming that they are linear systems. The framework can, for example, be used to guide the development of PCDs and PCD-based systems. Furthermore, it can be applied to adapt commonly used measures, such as the modulation transfer function, to non-linear PCDs.

## 1 Introduction

Photon-counting detectors (PCDs) herald the next leap in medical X-ray imaging, promising images with increased contrast-to-noise ratios, hence allowing for lower dose, and multi-energy imaging capabilities beyond what can be offered by dual-energy imaging,^1^^–^^3^ all of which could translate into substantial improvements in patient care. PCDs could therefore supersede the currently dominant energy-integrating detectors (EIDs) in X-ray imaging. Whereas EIDs only measure the total amount of energy deposited by X-rays in each projection, PCDs are capable of counting the number of detector pulses generated by individual X-ray photons and assigning each pulse to one of at least two energy bins. To compare and optimize the performance of different PCDs, suitable detector performance measures are needed. In particular, performance measures that have been devised for EIDs in the past may need to be adapted to the case of PCDs.

A fundamental aspect of any imaging detector is its transfer of the spatial and spectral information contained in the incident beam into its output signal. The corresponding measure is the detector point spread function (PSF), which, in our case, describes the detector’s response to irradiating a single pixel homogeneously.^4^ For PCDs, the PSF should not only describe the spatial response of the detector (i.e., how the incoming spatial information is distributed over neighboring pixels), but also the spectral response (i.e., how the incoming spectral information is distributed over energy bins).

An EID has, in very good approximation, a linear response. Hence, the response to an incident beam with an arbitrary spectrum can always be expressed as a linear combination of PSFs of monoenergetic beams. This also applies to a PCD under low fluence rate conditions; however, detectors are exposed to very high fluence rates of up to $3.5\cdot 10^{8}\;{\mathrm{mm}}^{-2}\;s^{-1}$ in clinical practice.^5^ Under these demanding circumstances, pile-up as well as the specific implementation of pulse processing will influence the transfer of spatial and spectral information and PCDs cease to exhibit a linear response.^6^^–^^8^ This warrants research into the characterization of PCDs under high fluence rates.

The aforementioned effect of *pile-up* is caused by the finite pulse length in any physical detector and refers to the overlap of individual pulses, such that a detector cannot determine the correct underlying number of events and their energies.^1^ We refer to the algorithm that is used to process the pulses and to extract the number of counts as well as the corresponding energies as the *counting behavior*. All in all, the finite pulse length of a detector in response to an incoming photon, the pile-up of pulses, and the subsequent readout via a specific counting behavior can lead to an incorrectly registered total number of photons, as well as to an incorrectly registered number of photons per energy bin. The extent of these effects is dependent on, first, the fluence rate, because a higher number of photons arriving at the detector per second and per detector pixel increases the pile-up probability, and second, the spectrum, because it determines the probability of an incoming photon of a specific energy to be detected in a certain energy bin after pile-up.

The research on non-linear effects in PCDs has so far focused on developing models to predict the counts registered in a particular energy bin^9^^–^^13^ and on calibrating for them,^14^^,^^15^ but less on the ramifications on commonly used tools to characterize detectors, such as the PSF, modulation transfer function (MTF), noise equivalent quanta (NEQ) and detective quantum efficiency (DQE), all of which are performance metrics based on the assumption of a linear detector response. We argue that a comprehensive characterization of PCDs must encompass their non-linear behavior and include the fluence rate into their analysis.

In this study, we explore how to adapt image quality measures to the non-linear response of PCDs. We demonstrate a basic example of calculating the contrast between two projection lines with slightly different line integrals, and show how an adapted formulation of the PSF, which we call perturbation PSF (pPSF), can be used in the non-linear regime. To illustrate the use of the newly proposed formalism, we show its application to simulated data of two PCDs by performing Monte-Carlo (MC) simulations and investigating the influence of the fluence rate of an X-ray beam with a given spectrum on the adapted detector measures. The focus of this work is on the introduction of a new theoretical framework, rather than on modeling any specific detector as realistically as possible. We therefore apply the framework to idealized versions of direct and indirect-conversion PCDs, intending to illustrate clearly how differences between detectors translate into differences in the pPSF.

## 2 Theory

### 2.1 Contrast for Single Projection Line

**Fig. 1:**
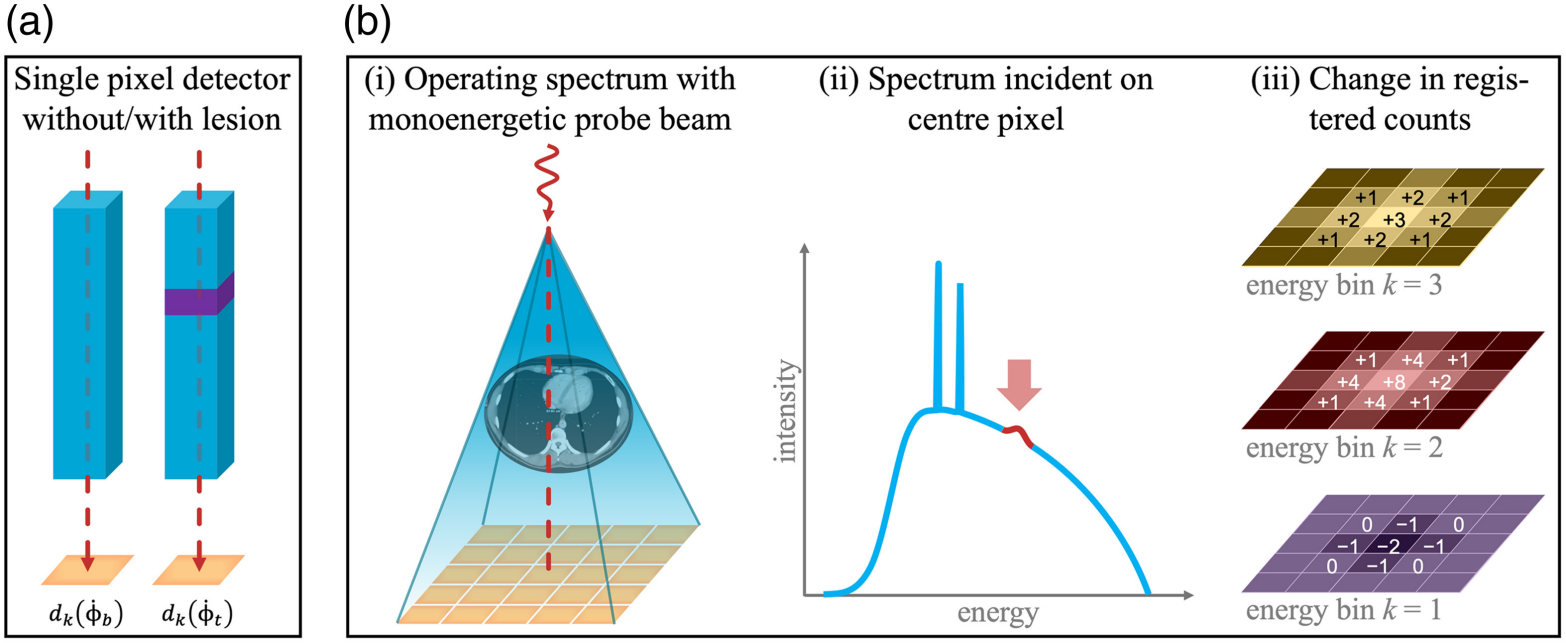
(a) A monoenergetic beam travels through an object, resulting in a registered count rate $d_{k}({\overset{˙}{\Phi }}_{b})$ in energy bin k of a single-pixel PCD. When a lesion is inserted, this results in a count rate $d_{k}({\overset{˙}{\Phi }}_{t})$. (b) Extension to a detector array and a polychromatic beam: (i) and (ii) A detector array is homogeneously irradiated by an operating spectrum (blue) with a given spectral fluence rate ${\overset{˙}{\Phi }}_{\mathrm{op}}$. This operating spectrum is perturbed by adding a monoenergetic probe beam (red) incident on the center pixel of the array. (iii) The change of count rate in the pixels and energy bins k of the PCD due to the addition of the monoenergetic perturbation can be approximated by a linear response.

Let us consider a PCD consisting of a single pixel, and a single projection line going through an object of interest [Fig. 1(a)]. The task is to distinguish between the cases of lesion absent and lesion present. We start with the measure of contrast, which is linked to the measure of detectability via the Rose criterion, which states a minimum required signal-to-noise ratio.^16^ Let $\overset{˙}{\Phi }=({\overset{˙}{\Phi }}_{E_{1}},\ldots ,{\overset{˙}{\Phi }}_{E_{L}})$ represent the incident spectral distribution, denoted as the spectral fluence rate, that is, the number of photons per unit area and unit time (${\mathrm{mm}}^{-2}\;s^{-1}$), where each entry represents the fluence rate at a discrete energy $E_{l}$. Here, discretization of the incoming photon energies is done for practical reasons; this facilitates the implementation of studies such as those described in Sec. 3. Let us furthermore denote the total fluence rate, that is, the total number of photons per ${\mathrm{mm}}^{2}$ per second, as ${\left\|\overset{˙}{\Phi }\right\|}_{1}=\underset{l}{\sum }{\overset{˙}{\Phi }}_{E_{l}}$.

For the case of a lesion being absent, that is, the baseline, we will denote the spectral fluence rate incident on the pixel by ${\overset{˙}{\Phi }}_{b}$, and the detector output, that is, the number of counts registered by the detector in energy bin k per unit time, by $d_{k}({\overset{˙}{\Phi }}_{b})$. Now let us insert a lesion, that is, the target, with a size small compared to the dimensions of the whole object, into the path of the projection line, slightly changing the fluence rate exiting the object. The spectral fluence rate on the detector shall now be denoted by ${\overset{˙}{\Phi }}_{t}$, and the number of registered counts in the pixel per time unit by $d_{k}({\overset{˙}{\Phi }}_{t})$. As an indication of how well the detector can distinguish between the two cases of lesion present and lesion absent, let us calculate the contrast $C_{k}$ between the two detector outputs in each detector energy bin k:

$$C_{k}=\frac{d_{k}({\overset{˙}{\Phi }}_{t})-d_{k}({\overset{˙}{\Phi }}_{b})}{d_{k}({\overset{˙}{\Phi }}_{b})}.$$ (1)

### 2.2 Small-Signal Analysis

To be able to make general statements about a system’s ability to preserve contrast, it is desirable to determine $d_{k}(\overset{˙}{\Phi })$ in Eq. (1) without having to actually measure it for every possible combination of total incident fluence rate and incident spectral shape. In case of a linear detector, this is straightforward if the detector response is known for every incident energy. To arrive at an expression for $d_{k}$ for the more challenging case of non-linear systems, we can assume that the spectral fluence rates for the cases with and without lesion only differ by a small amount, i.e., ${\overset{˙}{\Phi }}_{t}={\overset{˙}{\Phi }}_{b}+\Delta \overset{˙}{\Phi }$ with ${\left\|\Delta \overset{˙}{\Phi }\right\|}_{1}\ll {\left\|{\overset{˙}{\Phi }}_{b}\right\|}_{1}$, since we required the lesion to be small compared to the dimensions of the object. In other words, the insertion of the lesion is regarded as a perturbation $\Delta \overset{˙}{\Phi }$ of the original spectral fluence rate ${\overset{˙}{\Phi }}_{b}$. We can then Taylor-expand the registered count rate $d_{k}(\overset{˙}{\Phi })$ around the fluence rate ${\overset{˙}{\Phi }}_{b}$ up to and including first order, as follows:

$$d_{k}({\overset{˙}{\Phi }}_{t})=d_{k}({\overset{˙}{\Phi }}_{b}+\Delta \overset{˙}{\Phi })=d_{k}({\overset{˙}{\Phi }}_{b,E_{1}}+\Delta {\overset{˙}{\Phi }}_{E_{1}},\ldots ,{\overset{˙}{\Phi }}_{b,E_{L}}+\Delta {\overset{˙}{\Phi }}_{E_{L}})=d_{k}({\overset{˙}{\Phi }}_{b,E_{1}},\ldots ,{\overset{˙}{\Phi }}_{b,E_{L}})+\underset{l}{\sum }\frac{\partial d_{k}({\overset{˙}{\Phi }}_{b})}{\partial {\overset{˙}{\Phi }}_{E_{l}}}{\Delta \overset{˙}{\Phi }}_{E_{l}}+O(\Delta {\overset{˙}{\Phi }}^{2})\approx d_{k}({\overset{˙}{\Phi }}_{b})+\underset{l}{\sum }\frac{\partial d_{k}({\overset{˙}{\Phi }}_{b})}{\partial {\overset{˙}{\Phi }}_{E_{l}}}{\Delta \overset{˙}{\Phi }}_{E_{l}},$$ (2)

where ${\overset{˙}{\Phi }}_{b/t}=({\overset{˙}{\Phi }}_{b/t,E_{1}},\ldots ,{\overset{˙}{\Phi }}_{b/t,E_{L}})$ and $\Delta \overset{˙}{\Phi }=({\Delta \overset{˙}{\Phi }}_{E_{1}},\ldots ,{\Delta \overset{˙}{\Phi }}_{E_{L}})$ are vectors of discrete energies $E_{l}$. Inserting Eq. (2) into Eq. (1) then gives the contrast $C_{k}$ in energy bin k:

$$C_{k}=\frac{\underset{l}{\sum }\frac{\partial d_{k}({\overset{˙}{\Phi }}_{b})}{\partial {\overset{˙}{\Phi }}_{E_{l}}}{\Delta \overset{˙}{\Phi }}_{E_{l}}}{d_{k}({\overset{˙}{\Phi }}_{b})}.$$ (3)

**Fig. 2:**
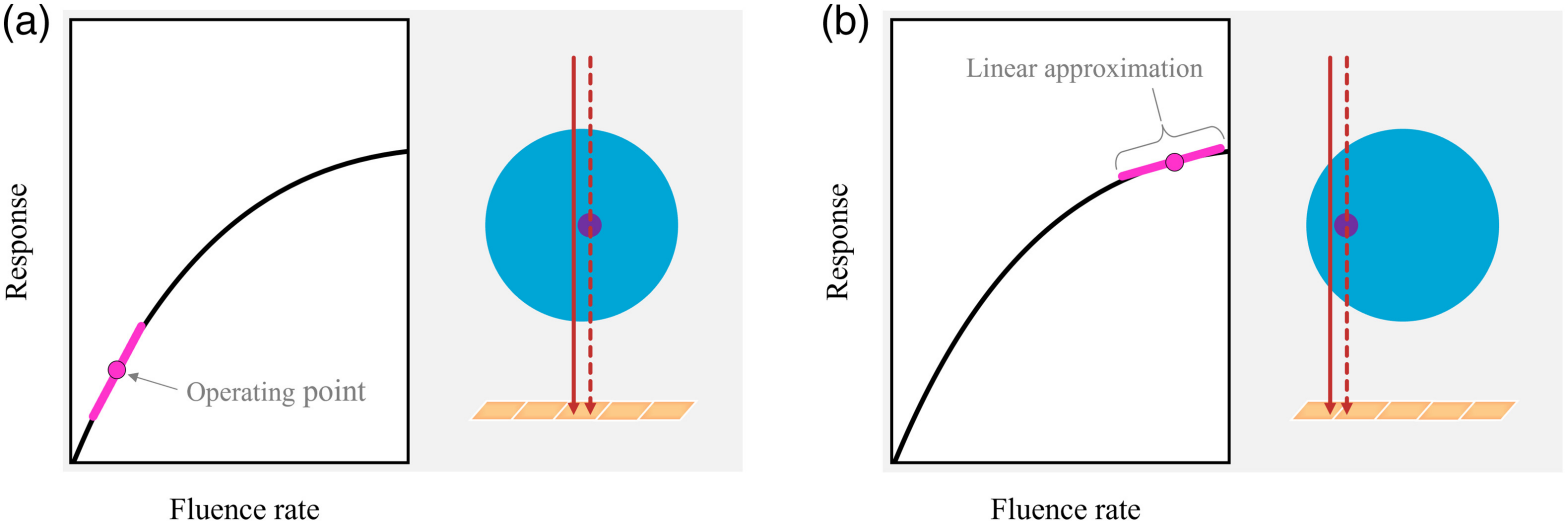
Schematic visualization of the proposed concept. A given fluence rate places the detector at a specific operating point. Around this operating point, the non-linear fluence rate—response curve is approximated as linear.

This expression describes contrast as the result of a sensitivity analysis, that is, how sensitive the detector is to a (small) change in fluence rate, given the baseline incident spectral fluence rate ${\overset{˙}{\Phi }}_{b}$. In essence, this is equivalent to approximating a non-linear system at a certain *operating point* as a linear system (Fig. 2). Therefore, we will denote the incident spectral fluence rate at which the detector is approximated by a linear system as the *operating spectrum* ${\overset{˙}{\Phi }}_{\mathrm{op}}$, which states the number of incident photons in units of ${\mathrm{mm}}^{-2}\;s^{-1}$. This idea of using small-signal analysis and a Taylor expansion has also been suggested (but not elaborated upon) by Tanguay et al.,^17^ while the terms “operating point”/“operating spectrum” follow the nomenclature commonly used in, e.g., electrical engineering, where one is interested in the behavior of a system at a specific operating point. The terms also underline the fact that the PCD’s response we aim to quantify is determined by the combination of all parameters and circumstances under which a detector is operated. One advantage of approximating a PCD as a linear system around a given operating point is that it enables us to study the change in detector response due to an arbitrary, though small, spectral fluence rate difference $\Delta \overset{˙}{\Phi }$. In the following section, we explore the gradient $\partial d_{k}(\overset{˙}{\Phi })/\partial {\overset{˙}{\Phi }}_{E_{l}}$ further.

### 2.3 Extension to Detector Arrays

So far, we have limited the discussion to a detector consisting of a single pixel. Here, we generalize the discussion to an array of pixels.

Following the notation introduced by Persson et al.,^18^ let us first introduce a detector PSF $h_{k}(n-n^{\prime },E_{l})$ which states the probability for photons of energy $E_{l}$, incident on the pixel $n^{\prime }$ with area A, to be registered as a count in pixel n and its energy bin k. To determine the PSF, a single pixel of the detector is illuminated and the response in all other pixels is registered. Under the assumption that the system is linear and shift-invariant, and given an incoming spectral fluence rate distribution $\overset{˙}{\Phi }(n)$, we can obtain the count rate $d_{k}$ in pixel n and energy bin k via [Persson et al.,^18^ p. 4899, Eq. (1)]

$$d_{k}(n,\overset{˙}{\Phi })=\underset{l}{\sum }\underset{n^{\prime }}{\sum }h_{k}(n-n^{\prime },E_{l}){\overset{˙}{\Phi }}_{E_{l}}(n^{\prime })\cdot A.$$ (4)

Equation (4) represents a (discrete) convolution of the incoming spectral fluence rate distribution $\overset{˙}{\Phi }(n)$ with the PSF $h_{k}$. This approach can be used to obtain the detector response of a linear detector to an arbitrary (spectral) fluence rate distribution. It is emphasized that Eq. (4) is only valid under the condition that the system is linear and shift-invariant, which in the case of PCDs is only fulfilled at low fluence rates.

At high fluence rates where the response of a PCD becomes non-linear a different approach is needed. We will start by assuming that the whole detector array is set at a specific operating point, that is, the detector array is irradiated homogeneously with a certain incident spectral fluence rate ${\overset{˙}{\Phi }}_{\mathrm{op}}$ [see Fig. 1b(i)]. This is motivated by the fact that the fluence rate behind an object is approximately constant within a sufficiently small region.

Next, we again approximate the non-linear system as a linear one around the chosen operating point and investigate the effect of a perturbation, which is realized using an additional probe beam of spectral fluence rate $\Delta {\overset{˙}{\Phi }}^{n_{0}}$ incident on pixel $n_{0}$ only [Figs. 1b(i) and 1b(ii)]. Since we approximate the non-linear system at the operating point ${\overset{˙}{\Phi }}_{\mathrm{op}}$ as a linear one, it is now possible to express the resulting change in the registered count rate $d_{k}$ as a convolution between $\Delta {\overset{˙}{\Phi }}^{n_{0}}$ and a PSF, similar to Eq. (4). The important distinction is that, in this case, the PSF must relate a *perturbation* of the incident fluence rate $\Delta {\overset{˙}{\Phi }}^{n_{0}}$ to a change in $d_{k}$ [Fig. 1b(iii)], and we will therefore denote it as the pPSF $h_{k}^{\Delta }(\Delta n,{\overset{˙}{\Phi }}_{\mathrm{op}},E_{l})$, with $\Delta n=n-n_{0}$. It must be stressed that $h_{k}^{\Delta }$ is bound to a specific operating point ${\overset{˙}{\Phi }}_{\mathrm{op}}$, and only applies to a small perturbation thereof.

Using the pPSF, we can express the registered count rate $d_{k}$ in pixel n as follows:

$$d_{k}(n,{\overset{˙}{\Phi }}_{\mathrm{op}}+\Delta {\overset{˙}{\Phi }}^{n_{0}})=d_{k}({\overset{˙}{\Phi }}_{\mathrm{op}})+\underset{l}{\sum }h_{k}^{\Delta }(n-n_{0},{\overset{˙}{\Phi }}_{\mathrm{op}},E_{l})\cdot {\Delta \overset{˙}{\Phi }}_{E_{l}}^{n_{0}}\cdot A.$$ (5)

Here we used that, since we assume a shift-invariant system and a constant fluence rate in a sufficiently small region, $d_{k}(n,{\overset{˙}{\Phi }}_{\mathrm{op}})$ is the same for all pixels n. Comparing Eq. (2) with Eq. (5) shows that the gradient $\partial d_{k}(\overset{˙}{\Phi })/\partial {\overset{˙}{\Phi }}_{E_{l}}$ corresponds to the pPSF.

## 3 Methodology

### 3.1 X-Ray Operating Spectrum

The operating spectrum assumed in this work is based on the standardized spectra described in the IEC 61267:2005 standard,^19^ more specifically, the set of so-called RQA spectra that mimic the X-ray beam behind a patient. The spectra are specified via a source voltage and an added aluminum filtration. Since realistic X-ray sources already exhibit intrinsic filtration, the standard specifies a nominal first half-value layer value to capture the spectrum’s shape in a single parameter. We simulated the spectra using SpekCalc^20^ with the source voltage and added filtration as defined in the IEC 61267:2005 standard. The intrinsic filtration was empirically adjusted to yield a very good agreement with the nominal first half-value layer for all spectra, and resulted in 0.8 mm Be and 0.10 mm Cu. In this work, we use the RQA9 spectrum as our operating spectrum, which is based on an X-ray tube voltage of 120 kVp.

### 3.2 Fluence Rates

For the choice of fluence rates relevant to medical imaging we refer to the work by Persson et al.^5^ In their study, they investigated the maximum fluence rates encountered in clinical CT protocols, using a CT scanner with tube current modulation and bowtie filter. The authors concluded that maximum total fluence rates between $3.4\cdot 10^{8}\;{\mathrm{mm}}^{-2}\;s^{-1}$ (standard head and chest protocol) and $4\cdot 10^{8}\;{\mathrm{mm}}^{-2}\;s^{-1}$ (ECG gated chest protocol) occur for perfectly centered patients. If the patients are misaligned, then the fluence rate can reach up to $6\cdot 10^{8}\;{\mathrm{mm}}^{-2}\;s^{-1}$. For non-standard protocols and maximum available tube currents on modern X-ray tubes, they may even reach up to $1.1\cdot 10^{9}\;{\mathrm{mm}}^{-2}\;s^{-1}$. For the present study, we therefore decided to cover a wide range of total fluence rates, from $10^{5}$ up to $10^{9}\;{\mathrm{mm}}^{-2}\;s^{-1}$ in increments of factors of 10.

### 3.3 Detector Models

This study considers two hypothetical PCDs, namely an idealized direct-conversion detector based on CdZnTe (CZT), and an idealized indirect-conversion detector based on a ${\mathrm{LaBr}}_{3}$:Ce scintillation crystal array one-to-one coupled to a silicon photomultiplier array. Indirect-conversion, scintillation-based PCDs may combine cost-effective growth of detector-grade material with efficient X-ray absorption, which is why silicon photomultiplier (SiPM)-based scintillation detectors are under investigation as an alternative to direct-conversion detectors based on, e.g., CdTe or CZT.^21^^–^^24^

While we simulate realistic energy resolutions and pulse shapes (see Sec. 3.5), we omit the inclusion of charge sharing and light leakage for the direct and indirect detector, respectively. As a consequence, the crosstalk between pixels observed in our study is entirely due to X-ray scatter and X-ray fluorescence, which are different for the two materials. By limiting the number of physical processes that contribute to crosstalk, we aim to clearly illustrate how differences between detectors correlate with differences in the pPSF.

The thicknesses of the absorbing layers were set to 2 mm for CZT and to 2.8 mm for ${\mathrm{LaBr}}_{3}$:Ce. The thicknesses were chosen such that, for the incident RQA9 spectrum, the number of photons passing through the detector material without any interaction is the same for both detectors.

The pixel pitch was set to 500  μm for both detectors (both in x- and y-direction); to account for a smaller active pixel size of the indirect-conversion detector due to a reflective layer around the scintillation crystals, a 60  μm thick^25^ PTFE (Teflon, $(C_{2}F_{2}{)}_{n}$) reflector was included, which was wrapped around each pixel and reduces the effective pixel size by 120  μm while keeping the pixel pitch at 500  μm.

### 3.4 Monte-Carlo Simulations of Energy Deposition

A MC simulation was implemented with GATE (version 9.2),^26^ which utilizes the GEANT4 toolkit^27^ (version 11.0.0). It simulates the irradiation of a detector with X-rays of a given spectrum (see Sec. 3.1) and tracks, for each incident photon, in which pixels energy is deposited, as well as the amount of deposited energy. The emission of photons from the source is modeled according to a Poisson process, and the timestamps of their interactions with the detector are stored.

CZT was defined as ${\mathrm{Cd}}_{0.9}{\mathrm{Zn}}_{0.1}\mathrm{Te}$ with a density^28^ of $5.78\;g/{\mathrm{cm}}^{3}$. ${\mathrm{LaBr}}_{3}$:Ce(5%) was defined with a 1:3 ratio between La and Br, where 5% of the La ions are replaced^29^ by Ce; the density^29^ was set to $5.29\;g/{\mathrm{cm}}^{3}$.

The physics model used by the MC simulation was based on the *emstandard_opt4* option in GATE. X-ray fluorescence was enabled by setting the production cut to 10  μm for photons and to 1000  μm for electrons. The remaining physics settings were kept at their default values. All events were registered and stored, without rejecting events below a certain energy threshold.

### 3.5 Pulse Train

The simulated events were processed with a pulse train analysis to mimic the readout circuitry of a PCD. For a given set of simulated events of energy $E_{i}$ with time stamps $t_{i}$ (sampled with 1 ns resolution), the generated pulse train is a convolution of a series of delta pulses, i.e., $\underset{i}{\sum }E_{i}\cdot \delta (t-t_{i})$, with a pulse shape function p(t). Prior to this convolution, an energy blurring was applied to model the finite energy resolution of the system, which was set to 22.3% full width at half maximum (FWHM) at 59.5 keV for the scintillation detector based on previously obtained experimental results using a 100 MHz low-pass filter^24^ and to 8.0% FWHM at 59.5 keV for the direct-conversion detector.^30^ The energy dependence of the FWHM energy resolution was modeled according to an inverse square law behavior in both cases.

For the CZT detector, a Gaussian pulse shape p(t) with a FWHM of 14 ns was chosen.^31^ For the ${\mathrm{LaBr}}_{3}$:Ce detector, the applied pulse shape p(t) is the convolution of two exponentially decaying functions modeling the scintillation decay (16 ns for ${\mathrm{LaBr}}_{3}$:Ce)^32^ and the recharge time of the SiPM (7 ns).^24^

### 3.6 Counting Behavior

In this study, we implemented a paralyzable-like (P-like) and a non-paralyzable-like (NP-like) counting behavior^33^ (see Fig. S1 in the Supplementary Material), both with a peak detection time $\tau_{\mathrm{pd}}$ that is started with a positive threshold crossing (P-like counting) or the start of an analysis window $\tau_{\mathrm{np}}$ (NP-like counting). Within the peak detection time window $\tau_{\mathrm{pd}}$ the maximum signal height is determined, used as the registered energy, and the counter in the respective energy bin is increased by 1.

The trigger threshold was set to 20 keV; while this value is relatively small in the context of clinical CT, it reveals a lower boundary of the event rate a detector can handle. While realistic PCDs with energy-discriminating capabilities use only a small number of energy bins, we used a near-continuous binning with bin widths of 1 keV to make the effects at play more visible, with the lowest bin centered at 20 keV and the highest at 250 keV; registered counts outside those limits were discarded.

The length of the analysis window $\tau_{\mathrm{np}}$ (for the NP-like counting only) is chosen such that it is slightly larger than the time over threshold (ToT) of a pulse caused by the photons with the highest energy. This ensures that the counting algorithm outputs a count of exactly 1 for the highest-energy photon in the incident spectrum in case there is no pile-up. The energy of the highest-energy photons is determined by the selected source voltage; since we include a limited energy resolution in our model, we increased this value by the FWHM of the energy resolution at this energy value. With the pulse shapes described in the previous section and for a 120 kVp RQA9 spectrum, this results into an analysis window of length $\tau_{\mathrm{np}}=51\;\mathrm{ns}$ for the ${\mathrm{LaBr}}_{3}$:Ce-based PCD and of $\tau_{\mathrm{np}}=24\;\mathrm{ns}$ for the CZT-based PCD. These values are very close to those reported by van der Sar et al.^24^ and Steadman et al.^31^

The peak detection time $\tau_{\mathrm{pd}}$ (for both NP- and P-like counting) is set slightly larger than the time between the trigger threshold crossing and the time where the pulse reaches its maximum. It is again determined for the photons with the highest energy, including an added energy blurring, and for photons of a 120 kVp RQA9 spectrum excluding any pile-up. This results into a peak detection time of $\tau_{\mathrm{np}}=11\;\mathrm{ns}$ for the ${\mathrm{LaBr}}_{3}$:Ce-based PCD, and of $\tau_{\mathrm{np}}=13\;\mathrm{ns}$ for the CZT-based PCD.

### 3.7 Determination of $h_{k}^{\Delta }$

Solving Eq. (5) for $h_{k}^{\Delta }$ yields

$$h_{k}^{\Delta }(\Delta n,{\overset{˙}{\Phi }}_{\mathrm{op}},E_{l})=\frac{d_{k}(n,({\overset{˙}{\Phi }}_{\mathrm{op},E_{1}},\ldots ,{\overset{˙}{\Phi }}_{\mathrm{op},E_{l}}+{\Delta \overset{˙}{\Phi }}_{E_{l}}^{n_{0}},\ldots ,{\overset{˙}{\Phi }}_{\mathrm{op},E_{L}}))-d_{k}(n,{\overset{˙}{\Phi }}_{\mathrm{op}})}{{\Delta \overset{˙}{\Phi }}_{E_{l}}^{n_{0}}\cdot A},$$ (6)

with A as the pixel area. Here, the perturbation in incident fluence rate is due to an added monoenergetic probe beam with fluence rate ${\Delta \dot{\Phi }}_{E_{l}}^{n_{0}}$ at energy $E_{l}$, with a cross section as large as one pixel, exclusively in pixel $n_{0}$. First, the registered count rate due to the operating spectrum, $d_{k}(n,{\overset{˙}{\Phi }}_{\mathrm{op}})$, was obtained as described in Secs. 3.1–3.6. Since we assume a shift-invariant detector homogeneously irradiated by the operating spectrum, $d_{k}(n,{\overset{˙}{\Phi }}_{\mathrm{op}})$ is the same for all pixels. Second, we added a monoenergetic probe beam with fluence rate ${\Delta \dot{\Phi }}_{E_{l}}^{n_{0}}$ at energy $E_{l}$ to pixel $n_{0}$ and stored the counts caused in all pixels n of the array. Third, $h_{k}^{\Delta }$ was calculated according to Eq. (6). The energy of the monoenergetic probe beam was swept from 20 to 150 keV in steps of 2 keV. For further details on the implementation of the evaluation of $h_{k}^{\Delta }$ we refer to Sec. S1.1 in the Supplementary Material.

### 3.8 Contrast and Contrast-to-Noise Ratio of Single Projection Line

Section 2.1 discusses the scenario of a single pixel detector measuring the spectral fluence rate behind an object with and without the insertion of a lesion. For the case of including a lesion, we attenuate the RQA9 spectrum with an additional object based on the Lambert–Beer law. The added lesion consists of an aqueous iodine solution with a concentration of 300  g/ml, defined with the following mass fractions: I: 0.2308; O: 0.6832; H: 0.0861; its energy-dependent mass attenuation coefficient μ(E)/ρ was extracted from the Xraydb library^34^ (version 4.4.7). The change in fluence rate due to the lesion is then calculated via ${\Delta \overset{˙}{\Phi }}_{E_{l}}={\overset{˙}{\Phi }}_{\mathrm{op},E_{l}}\cdot (\exp(-\mu (E_{l})/\rho \cdot x\rho )-1)$. The insertion of the lesion must be small enough that it does not change the (spectral) fluence rate significantly, hence, the thickness x and (homogeneous) mass density ρ of the lesion was selected such that the fluence rate in the energy range under investigation does not change by more than an arbitrary threshold of 1%. This is fulfilled by setting the product ρ·x to $0.0007\;g/{\mathrm{cm}}^{2}$.

The contrast was calculated according to Eq. (3). For the contrast-to-noise ratio (CNR), the contrast in each energy bin, $C_{k}$, was divided by the square root of the variance of the contrast in that energy bin, $\sigma (C_{k})$. The latter was determined via the variance of the number of counts in that energy bin, $d_{k}$. To obtain $\mathrm{Var}(d_{k})$, the pulse train was divided into shorter sections and the result for $d_{k}$ was saved for each section individually, which allowed to calculate the variance of $d_{k}$. The section length was adjusted such that the CNR was evaluated per equal dose for various fluence rates, i.e., for an increase in fluence rate by a factor of 10 the section length was decreased by a factor of 10. For further details on the determination of CNR we refer to Sec. S1.2 in the Supplementary Material.

## 4 Results

### 4.1 Perturbation Point Spread Function

**Fig. 3:**
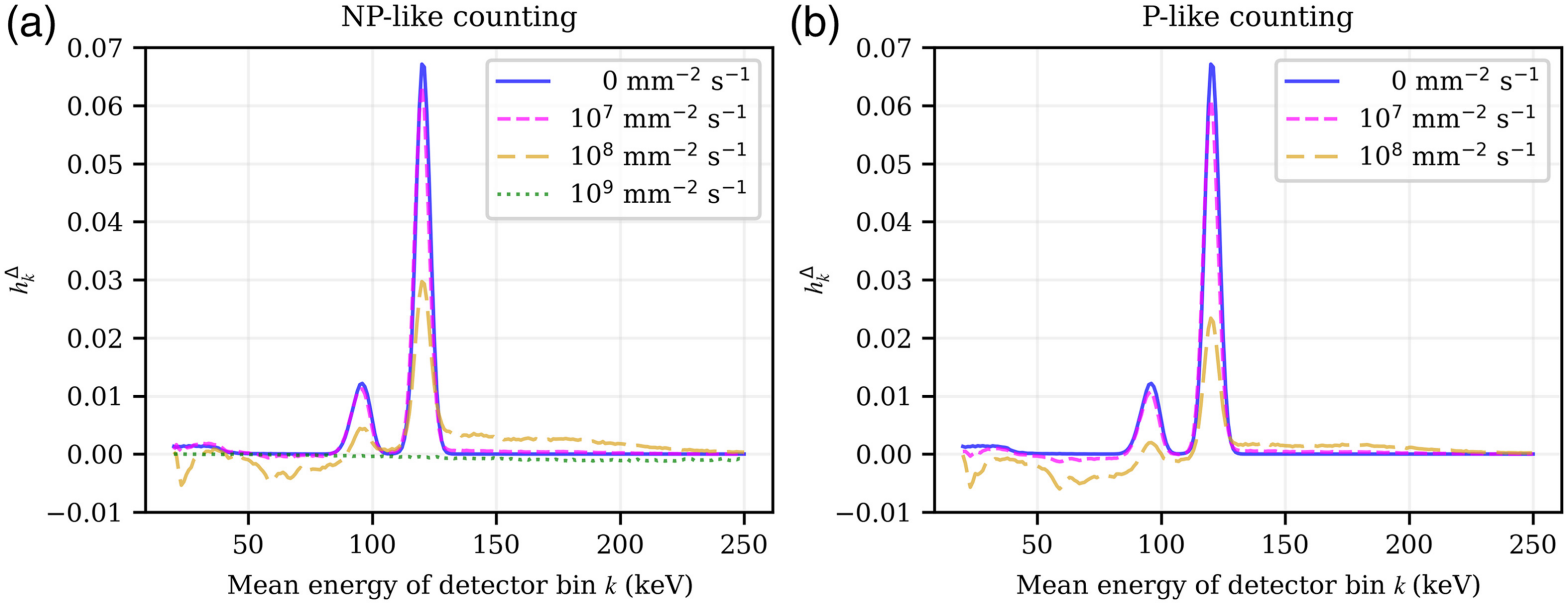
$h_{k}^{\Delta }(\Delta n=0,{\overset{˙}{\Phi }}_{\mathrm{op}},E_{l}=120\;\mathrm{keV})$, that is, a slice of the pPSF $h_{k}^{\Delta }$ along all registering energy bins k in the center pixel $n_{0}$ of the idealized direct-conversion detector for an incident probe beam energy $E_{l}$ of 120 keV. The figures show different total fluence rates of the operating spectrum ${\overset{˙}{\Phi }}_{\mathrm{op}}$, including the edge case of no operating spectrum, for (a) non-paralyzable and (b) paralyzable counting.

**Fig. 4:**
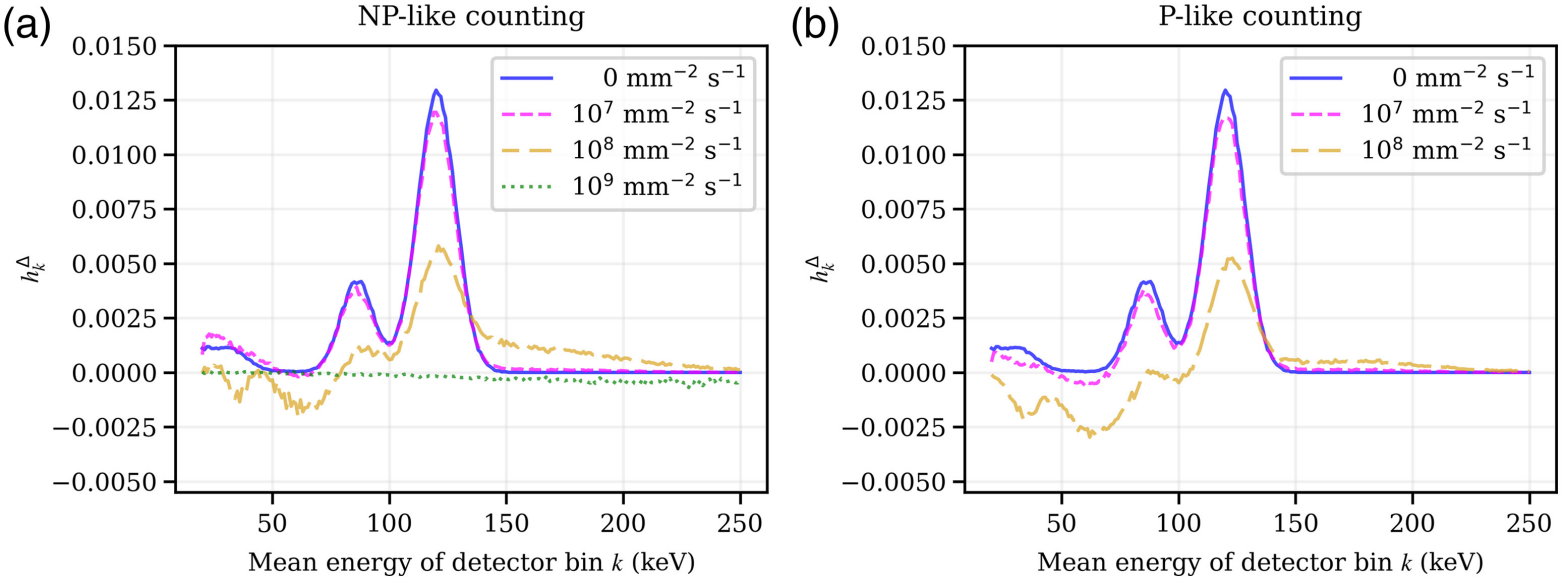
$h_{k}^{\Delta }(\Delta n=0,{\overset{˙}{\Phi }}_{\mathrm{op}},E_{l}=120\;\mathrm{keV})$, that is, a slice of the pPSF $h_{k}^{\Delta }$ along all registering energy bins k in the center pixel $n_{0}$ of the idealized indirect-conversion detector for an incident probe beam energy $E_{l}$ of 120 keV. The figures show different total fluence rates of the operating spectrum ${\overset{˙}{\Phi }}_{\mathrm{op}}$, including the edge case of no operating spectrum, for (a) non-paralyzable and (b) a paralyzable counting.

Figures 3 and 4 show $h_{k}^{\Delta }(\Delta n=0,{\overset{˙}{\Phi }}_{\mathrm{op}},E_{l}=120\;\mathrm{keV})$, that is, a slice of the pPSF $h_{k}^{\Delta }$ for the simulated idealized direct-conversion detector (iDCD) based on CZT and the idealized indirect-conversion detector (iICD) based on ${\mathrm{LaBr}}_{3}$:Ce, respectively, along all registering energy bins k in the center pixel $n_{0}$ (hence, Δn=0) for an incident probe beam energy $E_{l}$ of 120 keV. In other words, these graphs give the probability that an additional probe beam photon of energy $E_{l}$ adds (positive values) or removes (negative values) a count from a certain energy bin k. Note the difference with the PSF of linear systems, which is always positive. While realistic PCDs use only a few energy bins, the near-continuous binning shown here facilitates the understanding of the effects at play.

Figures 3 and 4 compare different fluence rates of the operating spectrum ${\overset{˙}{\Phi }}_{\mathrm{op}}$, including the edge case of no operating spectrum, under the assumption of a non-paralyzable (a) or a paralyzable (b) behavior, for the iDCD and iICD, respectively. For both the iCDC and iICD detector, the incoming probe beam is most likely registered in the energy bin k of the same energy, barring the limited energy resolution. Furthermore, a K-escape peak is clearly visible, located 23 to 26 keV (Cd)/27 to 31 keV (Te) below the main photopeak in the case of the iDCD, and 33 to 38 keV (La) in the case of the iICD. The iICD also features a higher probability of the incoming beam being registered in the energy bins below 50 keV.

For fluence rates of up to $10^{7}\;{\mathrm{mm}}^{-2}\;s^{-1}$, the detector responses change little compared to the limit of no operating spectrum being present. For higher fluence rates, however, the overall magnitude of $h_{k}^{\Delta }$ diminishes, which indicates an incipient saturation of the detector. Moreover, the incident probe beam starts to pile-up with the events already present in the operating spectrum, which means that higher energy bins may now register a count, while a count is at the same time removed from the low energy bins. As a consequence of the latter, the pPSF $h_{k}^{\Delta }$ can assume negative values, which can be prominently seen in case of the iICD detector under a fluence rate of $10^{8}\;{\mathrm{mm}}^{-2}\;s^{-1}$. For very high fluence rates of $10^{9}\;{\mathrm{mm}}^{-2}\;s^{-1}$ and a non-paralyzable behavior, both detectors completely saturate. Due to the limitations of our implementation to calculate the pPSF, the data for a fluence rate of $10^{9}\;{\mathrm{mm}}^{-2}\;s^{-1}$ and a paralyzable behavior are not shown (see Sec. S1.1 in the Supplementary Material for more information).

For a perfectly linear detector, i.e., a PCD with perfectly linear count rate behavior, $h_{k}^{\Delta }$ would have a value of 1 at the photopeak and 0 everywhere else. Since the iICD detector exhibits a smaller geometric efficiency due to a smaller effective pixel size (380  μm) compared to the pixels of the iDCD (500  μm), the absolute values for the probability of adding/removing a count due to an incoming photon of the probe beam (with a cross section of 500  μm×500  μm) are reduced compared to the iDCD.

**Fig. 5:**
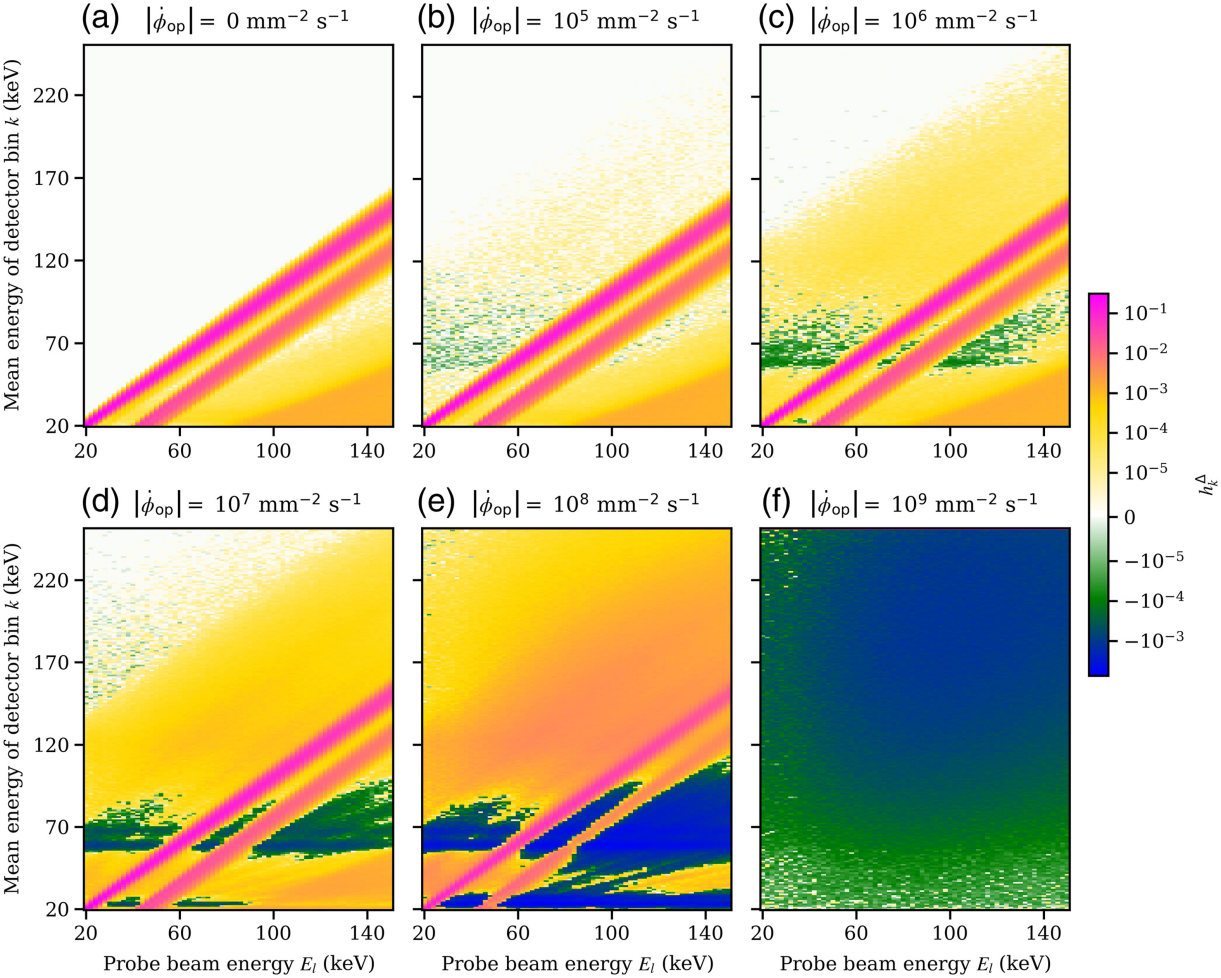
$h_{k}^{\Delta }(\Delta n=0,{\overset{˙}{\Phi }}_{\mathrm{op}},E_{l})$, that is, the pPSF $h_{k}^{\Delta }$ for all registering energy bins k and all simulated probe beam energies $E_{l}$ in the center pixel $n_{0}$ of the idealized direct-conversion detector. Here, a non-paralyzable behavior is assumed (see Fig. S3 in the Supplementary Material for the paralyzable case). (a)–(f) The results for various total fluence rates of the operating spectrum ${\overset{˙}{\Phi }}_{\mathrm{op}}$, starting from the edge case of no operating spectrum up to a fluence rate of $10^{9}\;{\mathrm{mm}}^{-2}\;s^{-1}$. The color scale is chosen such that it includes the global minimum and maximum values.

**Fig. 6:**
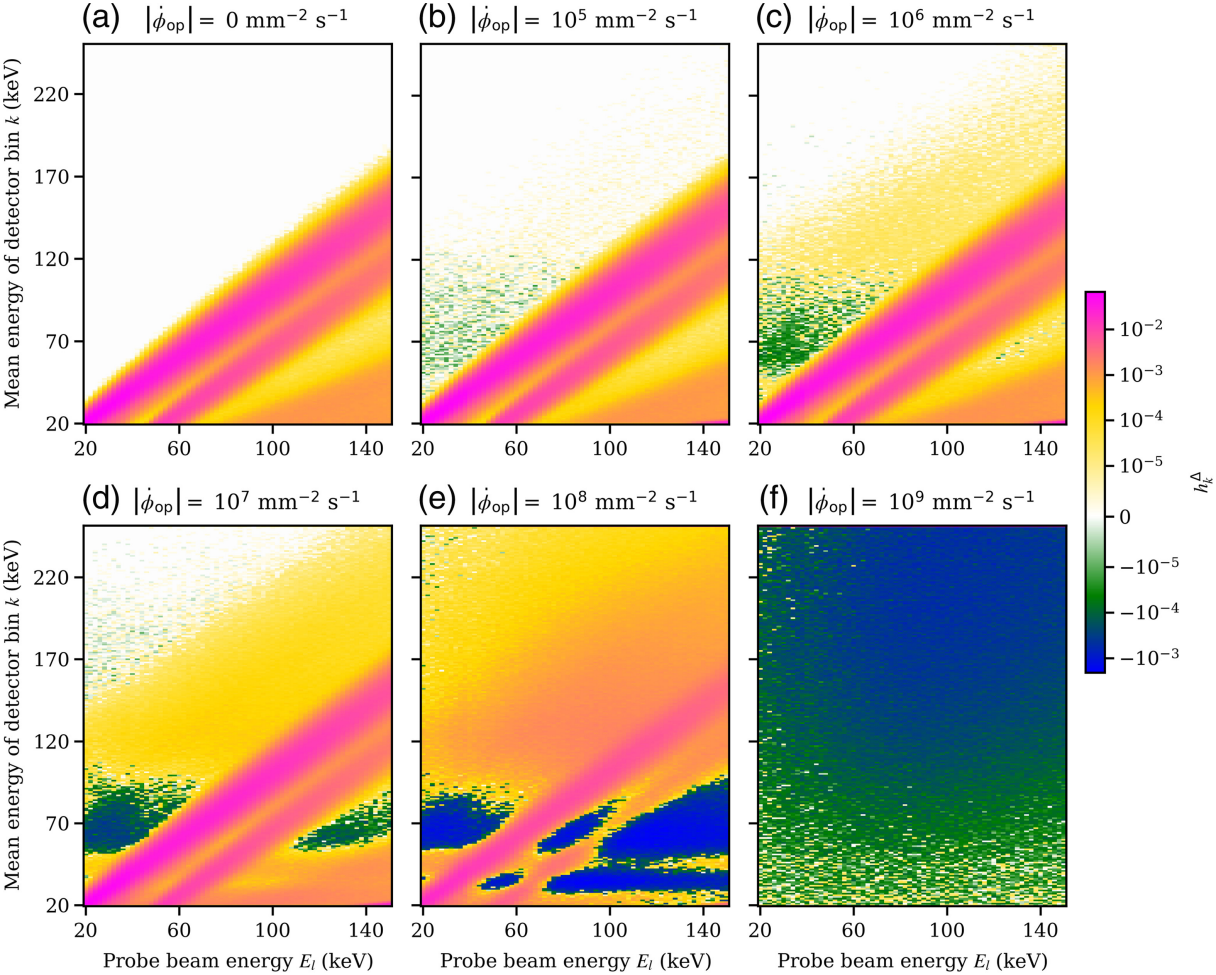
$h_{k}^{\Delta }(\Delta n=0,{\overset{˙}{\Phi }}_{\mathrm{op}},E_{l})$, that is, the pPSF $h_{k}^{\Delta }$ for all registering energy bins k and all simulated probe beam energies $E_{l}$ in the center pixel $n_{0}$ of the idealized indirect-conversion detector. Here, a non-paralyzable behavior is assumed (see Fig. S4 in the Supplementary Material for the paralyzable case). (a)–(f) The result for various total fluence rates of the operating spectrum ${\overset{˙}{\Phi }}_{\mathrm{op}}$, starting from the edge case of no operating spectrum up to a fluence rate of $10^{9}\;{\mathrm{mm}}^{-2}\;s^{-1}$. The color scale is chosen such that it includes the global minimum and maximum values.

**Fig. 7:**
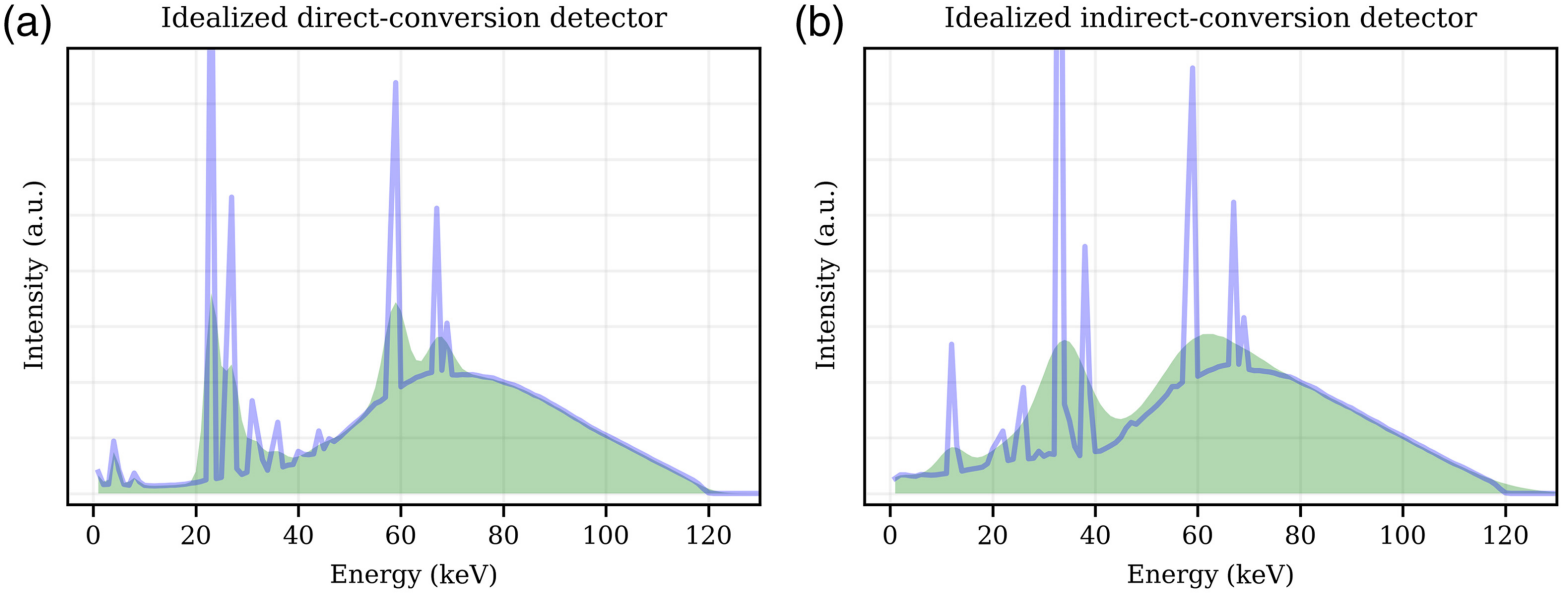
Energy spectra of deposited events (solid lines) in the idealized direct- (a) and indirect-conversion (b) detector, respectively, due to the incident operating spectrum. The filled curves show the spectra of deposited events when taking into account the limited energy resolution of the iDCD (8% at 59.5 keV) and the iICD (22.3% at 59.5 keV). The standardized RQA9 spectrum serves as the operating spectrum. Note that the figures show the spectra of deposited events (with and without a given energy resolution), not the spectrum registered by a detector for a given total fluence rate of the operating spectrum.

While Figs. 3 and 4 show a profile of $h_{k}^{\Delta }$ for a single incident probe beam energy $E_{l}$ of 120 keV, Figs. 5 and 6 show $h_{k}^{\Delta }(\Delta n=0,{\overset{˙}{\Phi }}_{\mathrm{op}},E_{l})$ for the iDCD and iICD, respectively; that is, $h_{k}^{\Delta }$ of the center pixel $n_{0}$ for all tested incoming energies $E_{l}$ and all registering energy bins k. The total fluence rates ${\left\|{\overset{˙}{\Phi }}_{\mathrm{op}}\right\|}_{1}$ of the operating spectrum vary from 0 [no operating spectrum present, (a)] to $10^{9}\;{\mathrm{mm}}^{-2}\;s^{-1}$ (f), assuming a paralyzable behavior. The two (pink) diagonal features in each figure (a)–(e) represent the photopeak and the accompanying K-escape peak, which are both broader in the iICD case due to the worse energy resolution. For fluence rates of the operating spectrum ${\left\|{\overset{˙}{\Phi }}_{\mathrm{op}}\right\|}_{1}>0$, we can see that the incoming probe beam piles up with events in the operating spectrum and hence causes counts in energy bins above the incoming probe beam energy. For both the iDCD and iICD, horizontal (green/blue) features are present with a high probability of removing counts [see for example Fig. 5(d)]. To understand these, the reader is referred to Fig. 7, which shows the energy spectra of deposited events in the iDCD (a) and iICD (b), respectively, due to the incident RQA9 operating spectrum. It should be stressed that Fig. 7 shows the spectra of deposited events with (filled curves) and without (solid lines) a given energy resolution, but not the spectrum registered by a detector for a given total fluence rate of the operating spectrum. A comparison of the spectra of deposited events (Fig. 7) with the figures for $h_{k}^{\Delta }$ (Figs. 5 and 6) reveals that the probability for removing events, i.e., the minima of $h_{k}^{\Delta }$, is the highest in those energy bins where the operating spectrum exhibits local maxima, i.e., creates most events, as these are most likely to pile up with probe beam events.

For a discussion on the spatial component of $h_{k}^{\Delta }$ along a row of detector pixels we refer to Sec. S2.1 in the Supplementary Material.

### 4.2 Contrast and Contrast-to-Noise Ratio for Single Projection Line

Section 2.1 discussed how to obtain the contrast of a non-linear PCD when inserting a lesion with thickness x and mass density ρ along the projection. For comparison, in a perfectly linear PCD, the registered count rate in a certain energy bin is proportional to the total fluence rate ${\left\|{\overset{˙}{\Phi }}_{\mathrm{op}}\right\|}_{1}$ of the incoming operating spectrum, i.e., $d_{k}({\overset{˙}{\Phi }}_{\mathrm{op}})∼{\left\|{\overset{˙}{\Phi }}_{\mathrm{op}}\right\|}_{1}$, and the absolute change of count rate due to an absolute change in the incoming spectrum, $\partial d_{k}({\overset{˙}{\Phi }}_{\mathrm{op}})/\partial {\overset{˙}{\Phi }}_{E_{l}}$, is constant irrespective of the fluence rate ${\left\|{\overset{˙}{\Phi }}_{\mathrm{op}}\right\|}_{1}$. Furthermore, in a perfectly linear PCD each event is registered with its original energy. As a consequence, given our example (Sec. 3.7), the contrast of a perfectly linear PCD is simply proportional to $\Delta \overset{˙}{\Phi }={\left\|{\overset{˙}{\Phi }}_{\mathrm{op}}\right\|}_{1}\cdot (\exp(-\mu (E)\cdot x)-1)$ according to Eq. (3), that is, the contrast follows the energy dependency of the X-ray attenuation of the lesion.

**Fig. 8:**
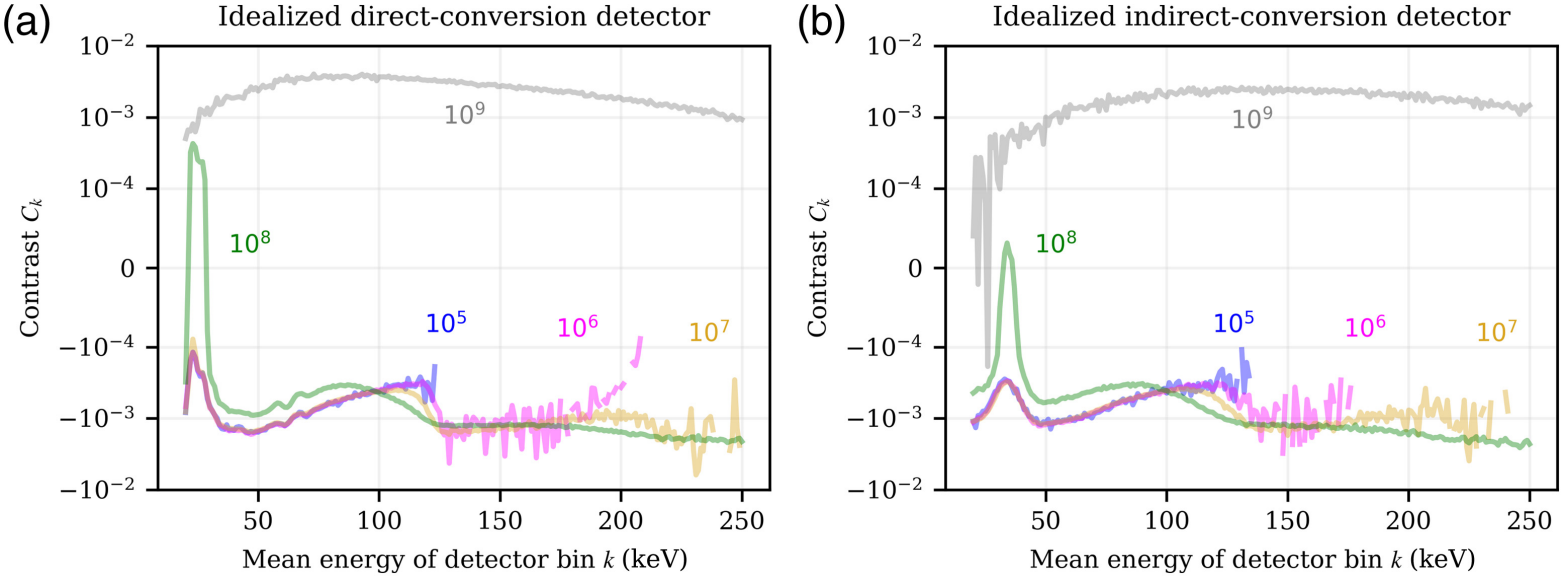
Contrast $C_{k}$ due to the insertion of a small water/iodine lesion, with a product of the lesion’s mass density and thickness of $\rho \cdot x=0.0007\;g/{\mathrm{cm}}^{2}$. The contrast is shown over the registering energy bins k of 1 keV width and for incident total fluence rates of the operating spectrum ${\overset{˙}{\Phi }}_{\mathrm{op}}$ between $10^{5}$ and $10^{9}\;{\mathrm{mm}}^{-2}\;s^{-1}$; the numbers indicate the total fluence rate corresponding with each curve in ${\mathrm{mm}}^{-2}\;s^{-1}$. For both the idealized direct- (a) and indirect-conversion (b) detector a non-paralyzable-like behavior was assumed. Missing data points in energy bins above 120 keV are due to division by zero in Eq. (3).

This result differs considerably from the behavior of more realistic PCDs shown in Fig. 8, based on the insertion of a water/iodine lesion as detailed in Sec. 3.8. It shows the contrast $C_{k}$ calculated according to Eq. (3), where the derivative $\partial d_{k}(\overset{˙}{\Phi })/\partial {\overset{˙}{\Phi }}_{E_{l}}$ in Eq. (3) corresponds to $h_{k}^{\Delta }(\Delta n,{\overset{˙}{\Phi }}_{\mathrm{op}},E_{l})\cdot A$. The figure depicts $C_{k}$ in each detector energy bin k for total fluence rates ${\left\|{\overset{˙}{\Phi }}_{\mathrm{op}}\right\|}_{1}$ of the operating spectrum between $10^{5}$ and $10^{9}\;{\mathrm{mm}}^{-2}\;s^{-1}$, assuming a non-paralyzable behavior for both the iDCD (a) and iICD (b) detector (see Figs. S5 and S6 in the Supplementary Material for the denominator and numerator of Eq. (3), respectively), and again a near-continuous binning with bin widths of 1 keV.

Several observations can be made in Fig. 8 for low to moderate total fluence rates of up to $10^{7}\;{\mathrm{mm}}^{-2}\;s^{-1}$. First, the contrast is negative since the fluence rate in the case with inserted lesion is smaller than in the case without lesion [Eq. (1)]. Second, the energy bins above the highest incident energy (120 keV in case of the RQA9 spectrum) exhibit on average a higher absolute value of contrast than those below. To understand this, we remind the reader that by definition the absolute value of contrast is highest for those bins in which a change in the incident fluence rate $\Delta \overset{˙}{\Phi }$ leads to the largest change in count rate. In the case of realistic PCDs with a finite pulse length and low to moderate fluence rate, most incoming photons will be registered in a bin of similar energy (deposition via photelectric effect) or smaller energy (K-escape, Compton/Rayleigh scattering); hence, the number of counts in those bins is already large (Fig. S5 in the Supplementary Material) and a change in fluence rate by $\Delta \overset{˙}{\Phi }$ yields only a small relative change in the number of counts in these bins. Due to the low probability of pile-up, however, there is a smaller number of counts in energy bins above the maximum incident energy to begin with; hence, a small absolute change in the number of pile-up events still translates to a large relative change in the number of counts in those bins, which in turn leads to a substantial increase in contrast.

Figure 8 furthermore shows that for high fluence rates larger than $10^{7}\;{\mathrm{mm}}^{-2}\;s^{-1}$ the contrast starts to switch signs. For example, the contrast at a fluence rate of $10^{8}\;{\mathrm{mm}}^{-2}\;s^{-1}$ exhibits both positive and negative values, depending on the energy bin. While Fig. 8 is based on a hypothetical PCD with bin sizes of 1 keV, a realistic PCD exhibits bin sizes in the order of tens of keV. As a consequence, the resulting contrast in such a realistic macro energy bin might be reduced (contrast cancellation) or even inverted (contrast inversion) compared to the contrast at low fluence rates, where the exact extent of this effect depends on the specific location of the energy bin boundaries.

Last but not least, for a very high fluence rate of $10^{9}\;{\mathrm{mm}}^{-2}\;s^{-1}$ the severe pile-up will cause most events to be registered with an energy above 250 keV and concurrently a significant reduction in the number of counts for energy bins below 250 keV. As a result, the denominator in Eq. (3) becomes very small, and in turn the absolute value for the contrast becomes very large.

**Fig. 9:**
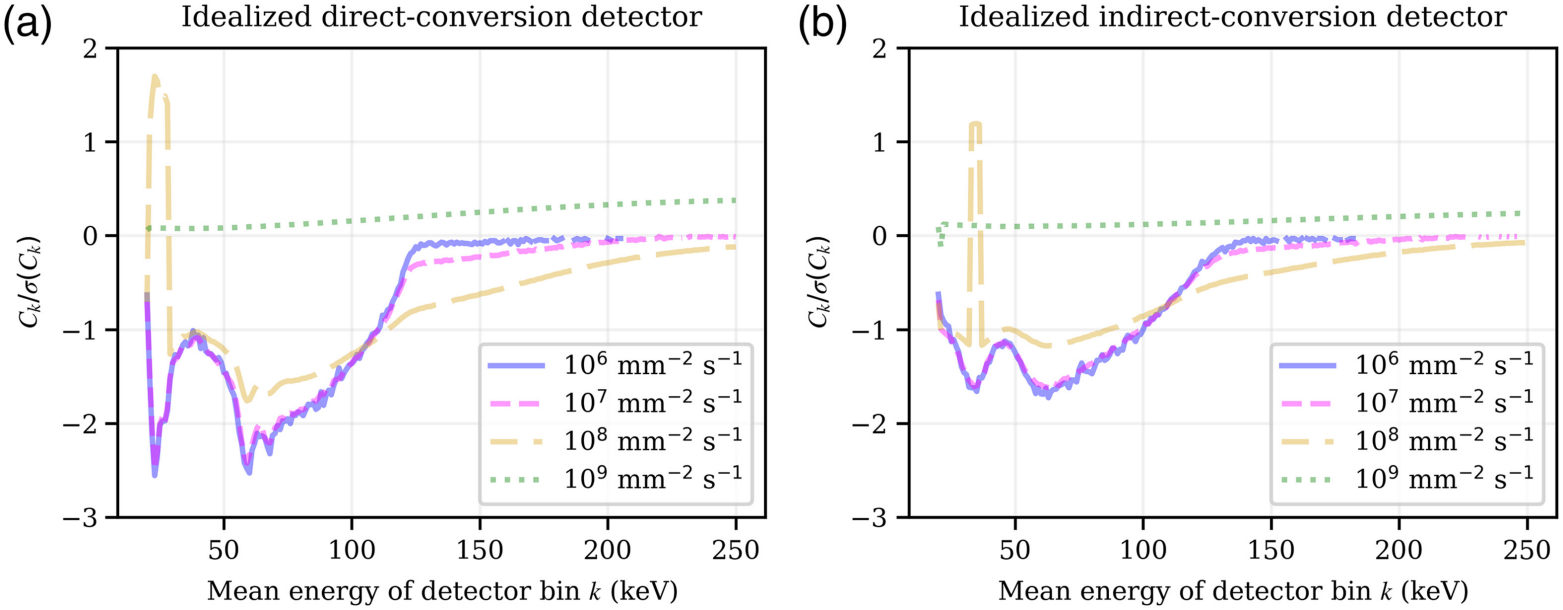
Contrast-to-noise ratio $C_{k}/\sigma (C_{k})$ due to the insertion of a small water/iodine lesion, with a product of the lesion’s mass density and thickness of $\rho \cdot x=0.0007\;g/{\mathrm{cm}}^{2}$. The CNR is shown over the registering energy bins k of 1 keV width and for incident total fluence rates of the operating spectrum ${\overset{˙}{\Phi }}_{\mathrm{op}}$ between $10^{6}$ and $10^{9}\;{\mathrm{mm}}^{-2}\;s^{-1}$. For both the idealized direct- (a) and indirect-conversion (b) detector a non-paralyzable-like behavior was assumed. Missing data points in energy bins above 120 keV are due to division by zero in Eq. (3).

This result may conflict the intuition that a detector should perform worse under conditions of severe pile-up. The cause for this discrepancy is that the above discussion was restricted to contrast only, whereas a clinically more relevant measure would be the CNR, which is shown in Fig. 9. It shows the CNR, $C_{k}/\sigma (C_{k})$, of various fluence rates calculated for equal amounts of dose, as described in Sec. 3.8, for both the iDCD and iICD. The comparison between the iDCD and iICD shows that the maximum CNR achieved by the former is about 50% higher than the CNR of the latter.

For energy bins below the highest incident energy, the absolute CNR value decreases with increasing fluence rate, whereas for energy bins higher than the highest incident energy the absolute CNR value increases with increasing fluence rate due to an increasing probability of pile-up. Furthermore, while the contrast at a fluence rate of $10^{9}\;{\mathrm{mm}}^{-2}\;s^{-1}$ exhibited the maximum absolute values, the CNR of the same fluence rate has now the smallest absolute values compared to all other fluence rates. The previous findings of contrast switching signs and the subsequent contrast reduction or contrast cancellation in realistic detectors with wider energy bins also translate to CNR.

For completion, while Figs. 8 and 9 show contrast and CNR, respectively, assuming near-continuous energy bins with widths of 1 keV, Sec. S2.2 in the Supplementary Material shows results using more realistic energy bin widths.

## 5 Discussion

The performance of the simulated idealized direct- and indirect-conversion detector shows similar trends across the presented results, with the differences mainly due to dissimilarities in energy resolution, pulse duration and geometric efficiency. For example, the CNR achieved by the iDCD in our example is about 50% higher compared to that of the iICD. In our setup, the iICD compensates its longer intrinsic pulse length with a reduced geometry efficiency due to the reflective septa between pixels; however, this in turn also reduces the dose efficiency.

Figures 8 and 9 show that, depending on the fluence rate, contrast/CNR cancellation or inversion may occur. For a paralyzable detector, this can be expected based on the count rate curve, which exhibits a maximum and therefore a point where its derivative and hence contrast becomes zero. However, Figs. 8 and 9 are for a non-paralyzable detector, which makes the explanation less intuitive: While the total count rate curve of a non-paralyzable detector is monotonically increasing, this is not necessarily true for the count rate curve of individual energy bins, which may see a decrease in counts at certain fluence rates due to pile-up. Complex behavior of the measured Hounsfield units over fluence rate has previously been reported by other authors,^6^^,^^35^ and we hope that our framework may help to shed light on the underlying mechanisms.

Since the focus of this study is on the introduction of a framework to characterize PCD performance in the presence of non-linear effects and not on achieving a high degree of accuracy in the simulation of any specific PCD, we omitted some effects that are important for the full understanding of the operating characteristics of realistic PCDs. For example, we omitted charge sharing and a potential charge summing circuitry in the case of the direct-conversion detector, as well as light leakage due to insufficient optical isolation in case of the indirect-conversion detector, all of which affect pixel crosstalk. Hence, the only causes of crosstalk between pixels observed in our study are X-ray scatter and X-ray fluorescence, which is equivalent to a pixel array with perfect physical isolation of pixels, and hence suppressing any charge or light sharing. While the commonly used CZT/CdTe detectors exhibit a continuous layer of semiconducting material and hence do not physically isolate neighboring pixels, an indirect-conversion detector array consisting of crystals with sufficiently thick septa, one-to-one coupled to an array of individual SiPMs and with perfect optical isolation, might come close to a detector without light leakage as modeled in our study.

We would like to add that, if charge sharing would be included for the direct-conversion detector, then based on the mean energy of 63 keV of the RQA9 background spectrum, an assumed diameter of the charge cloud^36^ of 29  μm, and our pixel size of 500  μm×500  μm, 11% of incoming events might be subject to charge sharing. This is only slightly less than the value of 18% obtained for a pixel size of 275  μm×322  μm, which is the pixel size used in the currently commercially available PCD CT scanners.^37^^,^^38^ While in both cases charge sharing would only affect a minority of events, theoretical studies^39^^–^^41^ have shown that the number of events subject to charge sharing does not always correlate linearly with the effects on the estimated quantity of interest. For example, in a study by Taguchi^41^ the variance on the estimate of the line integral of water in a water-bone material decomposition task was calculated for a direct-conversion PCD with and without charge sharing. Assuming a charge cloud diameter of 29  μm for the given 140 kVp spectrum, we estimate that in a 450  μm pixel about 12% of events might be subject to charge sharing, and about 24% for a 225  μm pixel. Taguchi showed that including the effect of charge sharing increases the variance on the line integral estimate by a factor of about 1.6 for a 450  μm pixel, whereas it increases by a factor of about 2.3 for a 225  μm pixel. For a full assessment of the effects of charge sharing in a specific detector on, e.g., contrast, CNR, or material decomposition, a detailed MC simulation study would be needed.

While charge sharing is often seen as an essential component for a realistic simulation of direct-conversion detectors, the simulation of light leakage may be considered an equally important component for the realistic simulation of indirect-conversion detectors in cases where the optical isolation between pixels is not perfect. If light leakage occurs, it leads to a different distortion of the registered spectrum compared to charge sharing: common charge sharing models for direct-conversion detectors assume a size of the charge cloud proportional to the incident energy,^36^ which leads to a larger proportion of high energy events affected by charge sharing. Furthermore, depending on the ratio between the charge cloud size and the pixel size, some events are not subject to charge sharing at all. In indirect-conversion detectors, on the other hand, the scintillation photons are emitted isotropically and the proportion of photons leaking into neighboring pixels is, at least in first-order approximation, independent of the location and energy of interaction, affecting all events similarly, potentially making this effect easier to correct for.

We would like to note that our framework itself is independent of the (number of) effects influencing detection that are taken into consideration, and it is merely a tool to investigate the results. Any effect influencing the registration of a count in a certain energy bin and pixel can be incorporated, as long as it can be attributed to a photon of an incoming probe beam. Our framework is most relevant, however, for characterizing PCDs in the presence of non-linear effects, which, in our study, are caused by pile-up and the specific implementation of the counting algorithm. In PCDs with charge summing circuitry, further non-linear effects can emerge under high fluence rates, as documented by Ji et al.^42^

While in this work we only studied the implications of pile-up on the measure of contrast and CNR, it seems feasible to apply the proposed framework of small-signal analysis to other commonly used measures of detector performance that are based on the PSF, such as the MTF or the frequency-dependent expressions for NEQ and DQE.^18^ However, it cannot be used to correct count rate curves, since our model is inherently based on a small-signal analysis.

In related research, Alvarez^43^^,^^44^ investigated the invertibility and condition number of transforming energy-resolved data into the line integrals of basis set coefficients of an n-material basis. He showed that the transformation may become ill-conditioned for specific line integral values in combination with certain energy spectra^41^ or in the presence of high pile-up.^43^ In this work, we focus solely on the effect of fluence rate on the detector itself keeping the line integral fixed, without considering the transformation to material basis coefficient line integrals, and show that this can already lead to contrast inversion. This approach may aid in better disentangling the different effects that influence the stability of material decomposition in PCDs.

With this new theoretical framework we aim to contribute to a better insight into the performance of PCDs under clinically relevant operating conditions. It can be used to quantify the detrimental effects of pile-up on spectral and spatial performance of PCDs for a given incident spectrum and fluence rate. Thus, the new framework can be used as a tool in the design, development, and characterization of PCDs and PCD-based systems.

## 6 Conclusions

In this work, we developed a framework to assess the non-linear spectral and spatial response of photon-counting detectors under pile-up conditions caused by high incident fluence rates. In the proposed framework small-signal analysis is employed, which approximates the non-linear behavior of PCDs by a linear response around a certain operating point and describes it by means of a so-called perturbation point spread function, which captures the spectral and spatial response of a PCD around that operating point. The operating point is determined by the spectral shape and the total fluence rate of the spectrum incident on the detector.

As an example, we showed how the pPSF can be used to determine the contrast and the contrast-to-noise ratio measured by a PCD for an arbitrary lesion in the projection path. The example illustrates the influence of pile-up on contrast and CNR, which may include non-intuitive effects such as contrast/CNR inversion or cancellation within an energy bin or between energy bins, and which supports the community’s efforts to achieve high intrinsic rate capabilities to avoid such effects in clinical images.

## Supplementary Material



## Data Availability

The data used in this work, the Python code used for processing and evaluating the data, the code for creating the result plots as well as the plotted data can be found here: https://doi.org/10.4121/8ce1a22f-16a4-450e-b24a-775278cad7a1.
