# Supplementary material for: Framework for evaluating photon-counting detectors under pile-up conditions
Source: J Med Imaging (Bellingham). 2024 May 24;11(Suppl 1):S12802. doi: 10.1117/1.JMI.11.S1.S12802 (PMC11124237; doi:10.1117/1.JMI.11.S1.S12802)
Supplement: Supplementary file 1 [file JMI_011_S12802_SD001.pdf]

A Framework for Evaluating Photon-Counting Detectors Under Pile-Up Conditions
# - Supplement -

David Leibold,[a] Stefan J. van der Sar,[a] Marlies C. Goorden,[a] Dennis R. Schaart[a,b]
[a]Department of Radiation Science and Technology, Delft University of Technology, Delft, The Netherlands
[b]Holland Proton Therapy Center, Delft, The Netherlands

S1 Supplementary Methodology

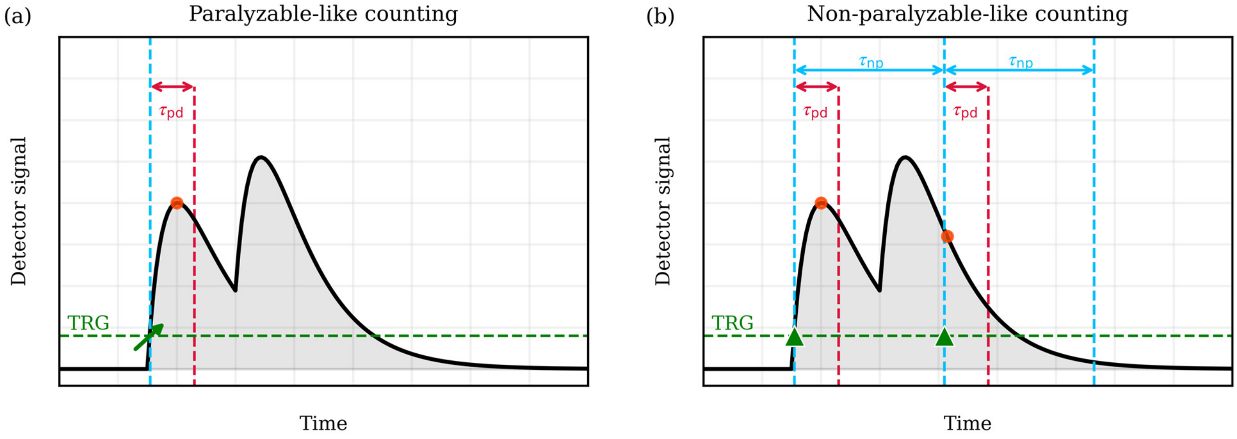

Figure S1: (a) Paralyzable-like behavior: After every positive trigger threshold crossing (green upward arrow) the maximum pulse height is determined (orange circle) within the peak detection time τ_{pd}. (b) Non-paralyzable-like behavior: If the signal is above the trigger threshold (green upwards triangles), an analysis window of length τ_{np} is started, and the maximum pulse height is determined (orange circles) within the peak detection time τ_{pd}. After the analysis window time elapsed, a new analysis window might start immediately.

S1.1 Determination of h_k^{Δ}

Section 3.7 of the paper describes how to determine h_k^{Δ}, which involves irradiating the pixel array with the operating spectrum and registering the count rate $d_k(\boldsymbol{n}, \dot{\boldsymbol{\Phi}}_{\mathrm{op}})$, as well as adding a monoenergetic probe beam to one pixel and registering the resulting count rate $d_k(\boldsymbol{n}, \dot{\boldsymbol{\Phi}}_{\mathrm{op}} + \Delta\dot{\boldsymbol{\Phi}}_{E_l}^{\boldsymbol{n}_0})$ in all other pixels (see Eq. (6)). This section will describe the implementation in more detail.

Fluence rate of the monoenergetic probe beam

The approximation of a non-linear detector response as linear around a certain operating point (such that it can be described via the pPSF h_k^{Δ}) is only valid as long as the perturbation by the probe beam is sufficiently small. For this, the fluence rate of the monoenergetic probe beam $\Delta\dot{\boldsymbol{\Phi}}_{E_l}^{\boldsymbol{n}_0}$ has to be low enough so that the likelihood of pile-up between events of the probe beam remains negligible, which ensures that each incident probe beam event can unambiguously be matched to a change in count rates. From this condition follows that the pile-up probabilities for $\dot{\boldsymbol{\Phi}}_{\mathrm{op}}$ and $\dot{\boldsymbol{\Phi}}_{\mathrm{op}} + \Delta\dot{\boldsymbol{\Phi}}_{E_l}^{\boldsymbol{n}_0}$ have to be approximately equal.

Efficient evaluation of h_k^{Δ}

Figure S2 schematically shows the idea of how to efficiently evaluate h_k^{Δ}. The straight forward approach to evaluate h_k^{Δ} while fulfilling the conditions to ensure linearity would be to dilate the probe beam pulse train (red) significantly (Fig. S2b, bottom row). However, this would mean that, in order to gain reasonable statistics, the pulse train to be evaluated would be prohibitively long.

Since the pulse train of the operating spectrum (blue) is not sufficiently long to cover the dilated probe beam pulse train, the operating spectrum could be repeated (Fig. S2b, top row). For a sufficiently dilated probe beam pulse train, the resulting scenario is equivalent to using the original operating spectrum pulse train once and adding each of the probe beam events one by one (Fig. S2c left, showing only one probe beam event). Fig. S2d shows a part of the resulting pulse train (pink) around the time stamp of the probe beam event (vertical dashed lines in Fig. S2c, right). In each iteration first the pulse train of the operating spectrum is evaluated with the selected counting behavior (resulting into $d_k(\boldsymbol{n}, \dot{\boldsymbol{\Phi}}_{\mathrm{op}})$), then the combined pulse train of the operating spectrum plus the pulse train is evaluated (resulting into $d_k(\boldsymbol{n}, \dot{\boldsymbol{\Phi}}_{\mathrm{op}} + \Delta\dot{\boldsymbol{\Phi}}_{E_l}^{\boldsymbol{n}_0})$), and the difference in the counts in all energy bins is saved.

However, evaluating the whole pulse train for just one added probe beam event is still computationally very expensive, especially considering the fact that most of the pulse train will stay unaffected by the addition of a single probe beam event. Hence, it is sufficient to only evaluate a small region around the probe beam's time stamp (Fig. S2c right, vertical dashed lines). In our implementation, we search for naturally occurring gaps in the operating spectrum pulse train (marked red in Fig. S2e), dividing the pulse train into smaller clusters. The size of the gaps is equal to the length of a pulse, sufficient that a single additional event added to one cluster is unable to affect the pulse train after two clusters and two gaps. This approach enables us to evaluate pairs of clusters separately, i.e., superimposing the monoenergetic probe beam events one by one and calculating h_k^{Δ} for one cluster for each added event. Two neighboring clusters (brackets in Fig. S2e) were combined for the evaluation of a single monoenergetic probe beam event. Monoenergetic events were added which occurred during the first cluster including the subsequent gap (solid part of the bracket); the second cluster (dashed part of the bracket) was included since

a monoenergetic probe beam event added to the first cluster (plus gap) could change the registered counts up to and including the second cluster, but not beyond the gap after the second cluster.

With this approach it is computationally feasible to determine h_k^{Δ} for a large number of monoenergetic probe beams and a large number of pixels. We validated our approach by comparing it to the straight-forward implementation shown in Fig. S2b.

In case the fluence rate of the operating spectrum was so high that no or only few and large clusters were formed, the pulse train was artificially split in order to accelerate the simulation; this was only necessary for a total fluence rate of 10^9 mm^{-2} s^{-1}. However, such artificial splitting introduces additional trigger events in the case of P-like counting, which appeared to be the only source of trigger events at this fluence rate. Hence, the data for P-like counting at a total fluence rate of 10^9 mm^{-2} s^{-1} was not included in the analysis of results.

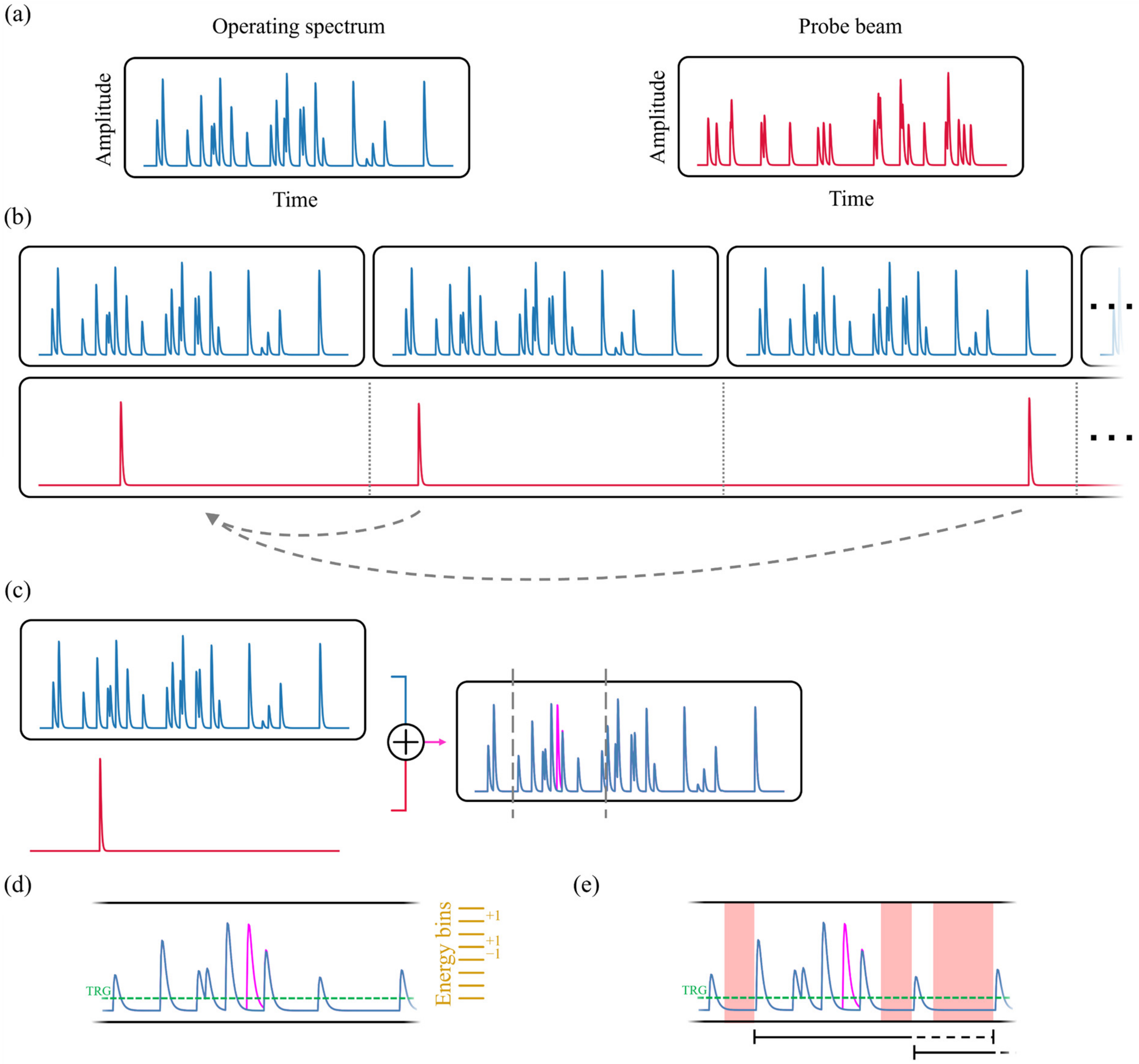

Figure S2: Schematic overview of the implementation to determine h_k^{Δ}. (a) Operating spectrum pulse train (blue) and probe beam pulse train (red). To ensure linearity when adding the probe beam (see text), the time stamps of the probe beam have to be dilated significantly ((b), bottom row). Since the pulse train of the operating spectrum pulse train is not sufficiently long to cover the dilated probe beam pulse train, the operating spectrum pulse train is repeated ((b), top row). The resulting scenario is equivalent to using the operating spectrum's original pulse train and adding each of the probe beam events one by one ((c), showing only one probe beam event). (d) First the pulse train of the operating spectrum is evaluated, then the combined pulse train of operating spectrum plus probe beam, and the difference in the counts in energy bins is saved. Since most of the pulse train will stay unaffected by the addition of a single probe beam event, it is sufficient to only evaluate a small region around the probe beam's time stamp. We therefore search for gaps in the pulse train (marked red in (e)) which are large enough to ensure that any change to the pulse train by adding one event within two gaps is unable to change the pulse train after the gaps. See text for further explanations.

S1.2 Variance of contrast

The variance of the contrast was calculated using error propagation. Starting from the formula for contrast,

$$C_k = \frac{\sum_l h_k^{\Delta}(\Delta \boldsymbol{n}=0,\ \dot{\boldsymbol{\Phi}}_{\mathrm{op}},\ E_l) \cdot \Delta\dot{\Phi}_{E_l} \cdot A}{d_k(\dot{\boldsymbol{\Phi}}_{\mathrm{op}})} \quad , \tag{S1}$$

the variance on C_k caused by the variance on d_k can be calculated as follows:

$$\mathrm{Var}(C_k) = \left(\frac{\partial C_k}{\partial d_k}\right)^2 \mathrm{Var}(d_k) \quad . \tag{S2}$$

Here, we neglected the influence by the change in fluence rate $\Delta\dot{\boldsymbol{\Phi}}$ on the variance, since, firstly, our investigation showed that this contribution to the variance of C_k is negligible compared to the variance due to d_k, and, secondly, the physical intuition is that, in order to determine the contrast-to-noise ratio, the variance is measured in a region consisting only of background, and the variance in this region is purely governed by the case that no lesion is present, i.e., Var(d_k).

Inserting Eq. (S1) into Eq. (S2) results in:

$$\mathrm{Var}(C_k) = \left(\frac{\sum_l h_k^{\Delta}(\Delta \boldsymbol{n}=0,\ \dot{\boldsymbol{\Phi}}_{\mathrm{op}},\ E_l) \cdot \Delta\dot{\Phi}_{E_l} \cdot A}{d_k^2(\dot{\boldsymbol{\Phi}}_{\mathrm{op}})}\right)^2 \mathrm{Var}(d_k) \quad , \tag{S3}$$

where Var(d_k) is equal to the mean of d_k. While this assumption holds only for Poisson-distributed data, it was checked that despite the non-linear behavior of a PCD the variance of d_k in our simulation study is indeed equal to its mean over the whole range of investigated fluence rates.

S2 Supplementary Results and Discussion

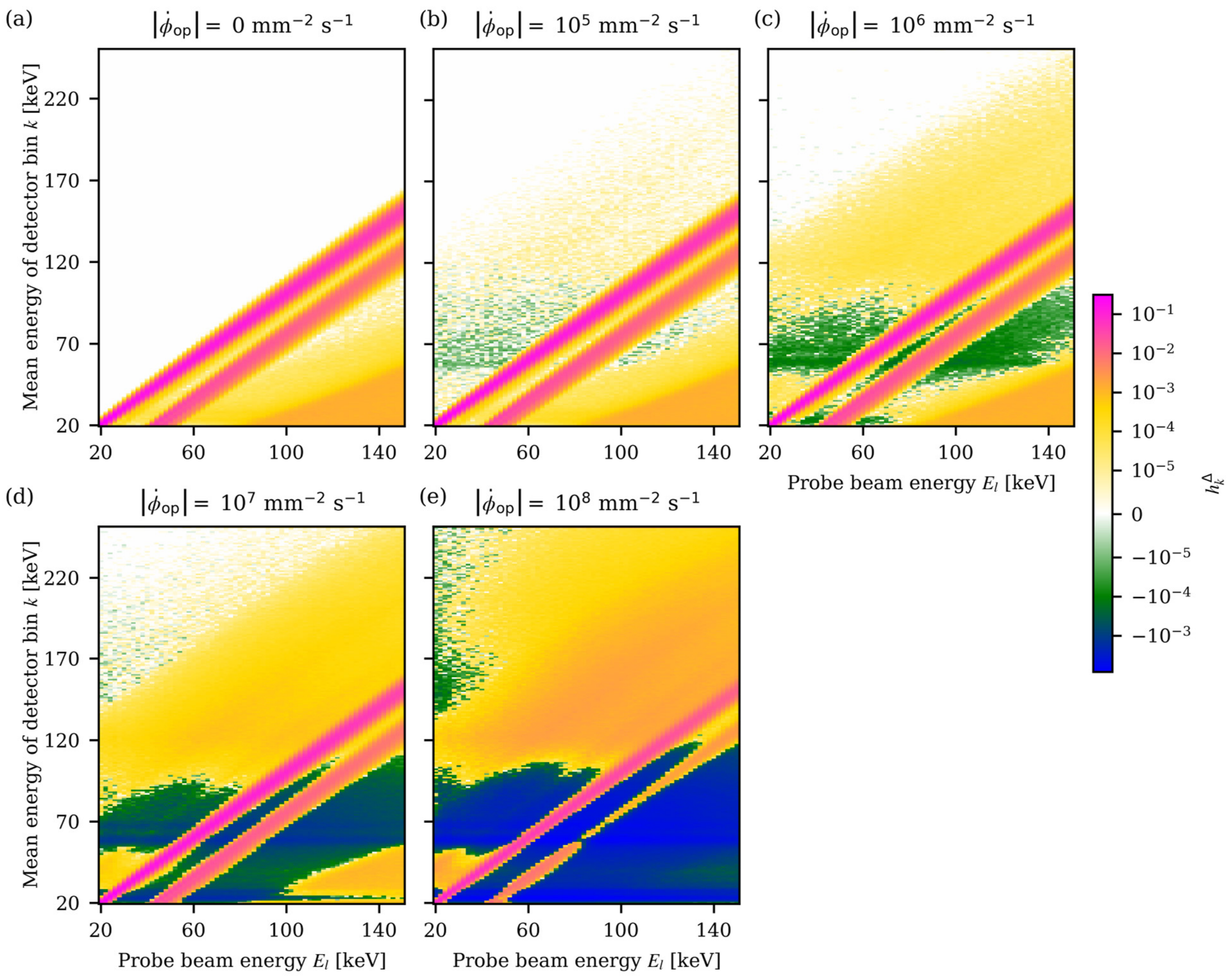

Figure S3: $h_k^\Delta(\Delta\boldsymbol{n} = 0, \dot{\boldsymbol{\Phi}}_{\mathrm{op}}, E_l)$, that is, the pPSF h_k^Δ for all registering energy bins k and all simulated probe beam energies E_l in the center pixel $\boldsymbol{n}_0$ of the idealized direct-conversion detector. Here, a paralyzable counting behavior is assumed. (a) – (f) show the result for various total fluence rates of the operating spectrum $\dot{\boldsymbol{\Phi}}_{\mathrm{op}}$, starting from the edge case of no operating spectrum up to a fluence rate of 10^9 mm^{-2} s^{-1}. The color scale is chosen such that it includes the global minimum and maximum values.

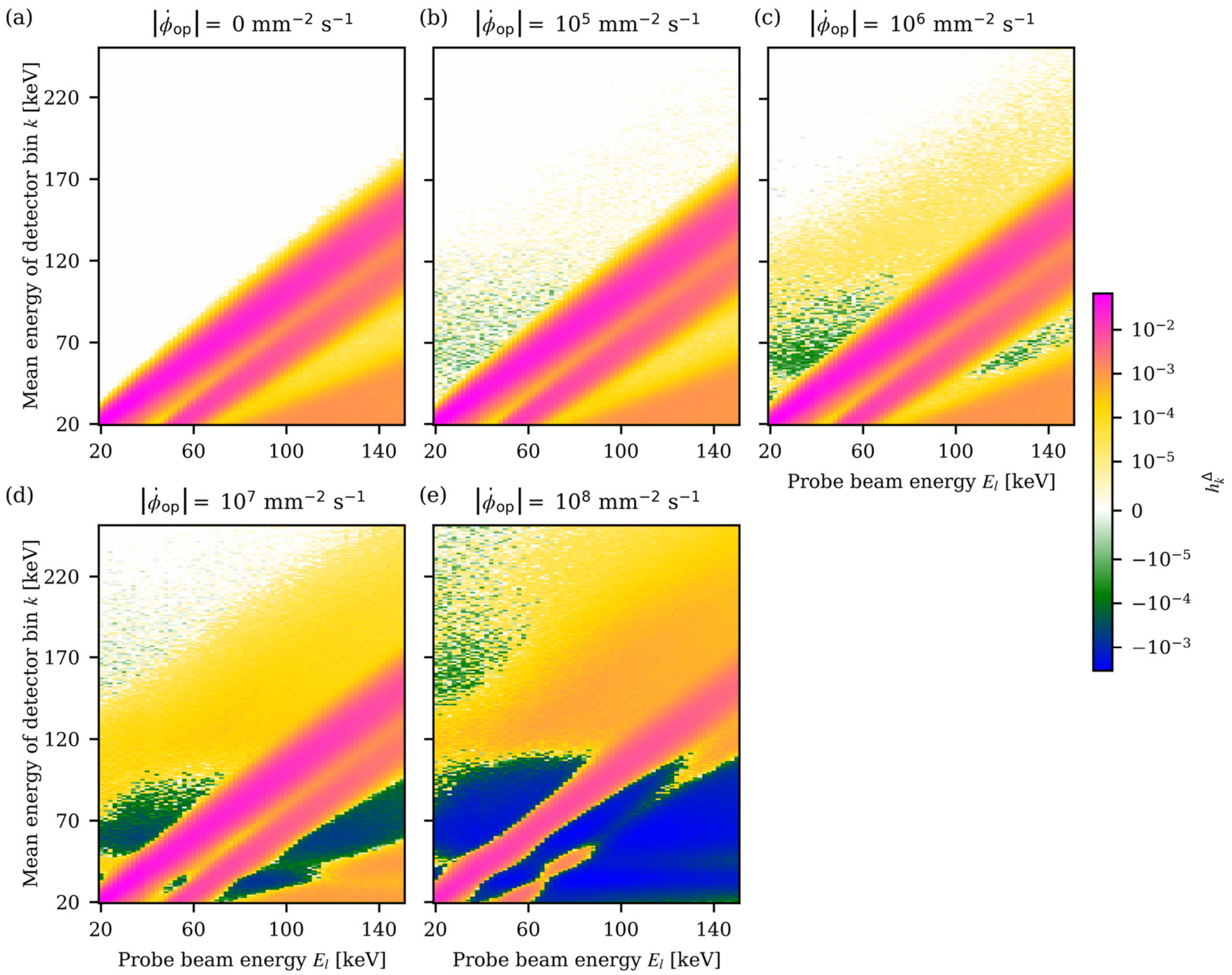

Figure S4: $h_k^\Delta(\Delta \boldsymbol{n} = 0, \dot{\boldsymbol{\Phi}}_{\mathrm{op}}, E_l)$, that is, the pPSF h_k^Δ for all registering energy bins k and all simulated probe beam energies E_l in the center pixel $\boldsymbol{n}_0$ of the idealized indirect-conversion detector. Here, a paralyzable counting behavior is assumed. (a) – (f) show the result for various total fluence rates of the operating spectrum $\dot{\boldsymbol{\Phi}}_{\mathrm{op}}$, starting from the edge case of no operating spectrum up to a fluence rate of 10^9 mm^{-2} s^{-1}. The color scale is chosen such that it includes the global minimum and maximum values.

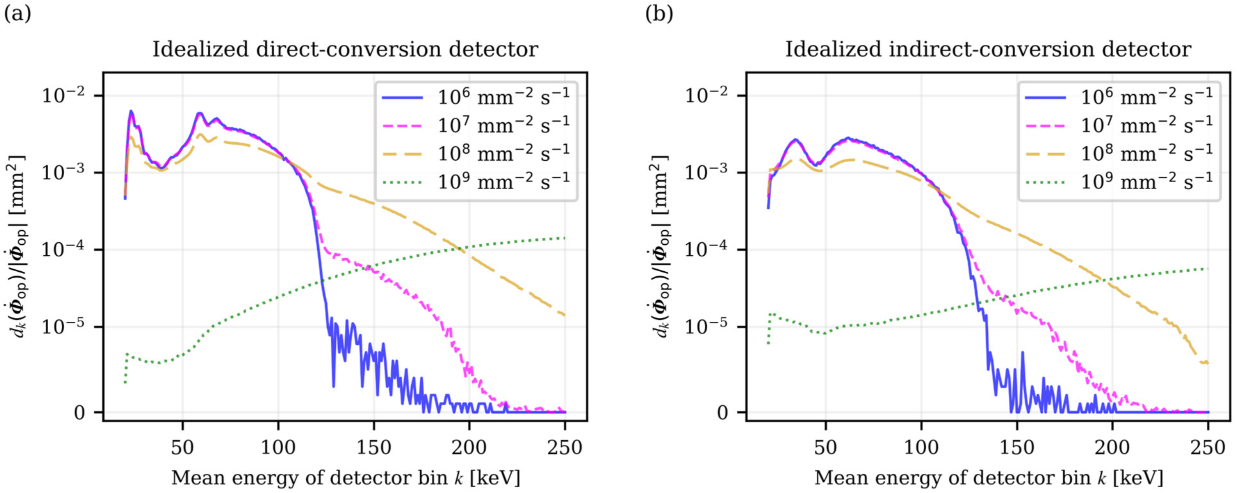

Figure S5: $d_k(\dot{\boldsymbol{\Phi}}_{\mathrm{op}})/\|\dot{\boldsymbol{\Phi}}_{\mathrm{op}}\|_1$, that is, the count rate in detector bin k of one pixel divided by the total fluence rate, for different fluence rates of the operating spectrum. (a) shows the result for the idealized direct-conversion detector, and (b) for the idealized indirect-conversion detector, both with a non-paralyzable counting behavior.

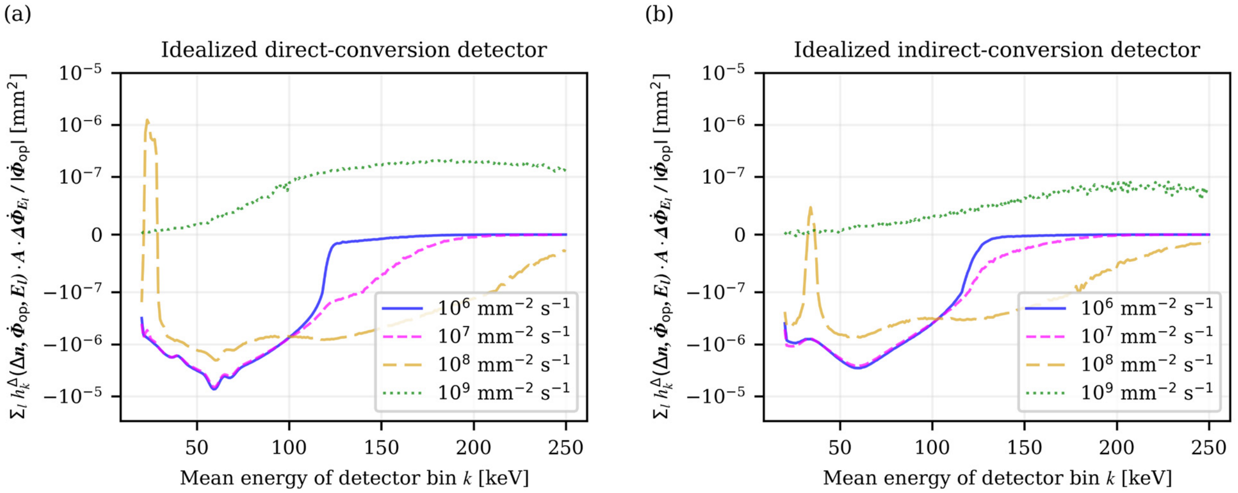

Figure S6: $\sum_l h_k^\Delta(\Delta\boldsymbol{n}, \dot{\boldsymbol{\Phi}}_{\mathrm{op}}, E_l)\cdot A\cdot\Delta\dot{\Phi}_{E_l}/\|\dot{\boldsymbol{\Phi}}_{\mathrm{op}}\|_1$, that is, the numerator of Eq. (3) divided by the total fluence rate, for different fluence rates of the operating spectrum. (a) shows the result for the idealized direct-conversion detector, and (b) for the idealized indirect-conversion detector, both with a non-paralyzable counting behavior. The water/iodine lesion is assumed to be characterized by $\rho\cdot x = 0.0007$ g/cm^2.

S2.1 h_k^{Δ} for a row of pixels

Figures S7 and S8 show how h_k^{Δ} varies for a row of pixels of a detector array, including the center pixel receiving the probe beam. Figure S7 (b – e) and Figure S8 (b – e) show $h_k^{\Delta}(\Delta\boldsymbol{n}, \dot{\boldsymbol{\Phi}}_{\mathrm{op}}, E_l)$, that is, the pPSF h_k^{Δ} for all registering energy bins k and all simulated probe beam energies E_l of the idealized direct-conversion and the idealized indirect-conversion detector, respectively. The schematic in Fig. S7a and S8a indicates the position of the pixels whose pPSF is shown. A paralyzable counting behavior and a total fluence rate of the operating spectrum $\dot{\boldsymbol{\Phi}}_{\mathrm{op}}$ of 10^6 mm^{-2} s^{-1} is assumed.

For the discussion of the pPSF of the center pixel we refer to Section 4 of the paper. As can be seen in Figures S7 (c – e) and S8 (c – e), the probe beam incident on the center pixel can be registered in the neighboring pixels via either K-fluorescence events, which explains the features around energy bins at 23 – 31 keV (Cd, Te) in Fig. S7 (c – e) and around 33 – 38 keV (La) in Fig. S8 (c – e), or via Compton/Rayleigh scatter events that preserve most of the initial photon's energy (diagonal features in (c – e)).

Figures S7f and S8f show how a probe beam with a white spectrum, i.e., all energies E_l are equally likely, is registered by a row of detector pixels centered around the center pixel receiving the probe beam, for various fluence rates. The plot then gives the probability that a photon of the incoming probe beam is detected in any energy bin of the respective pixel; this is analogous to summing $h_k^{\Delta}(\Delta\boldsymbol{n}, \dot{\boldsymbol{\Phi}}_{\mathrm{op}}, E_l)$ for each pixel and normalizing it by the number of energies in the probe beam. Due to the large pixel size of 500 µm, the probability of registering a count in one of the pixels adjacent to the center pixel decreases rapidly (please note the log scale of the plot). The probability of detecting a photon from the probe beam in any energy bin of a pixel stays mostly constant for all except the highest fluence rates. This is because for our idealized detectors the spatial

distribution of the deposition of energy is governed by X-ray transport only, and although an increase in pile-up due to an increased fluence rate of the operating spectrum changes the registered spectrum, most events are still registered in some energy bin. As a consequence, the probability for detecting a probe beam photon in any energy bin stays mostly constant for all except the highest fluence rates, for which the detector goes into saturation and counts are missed.

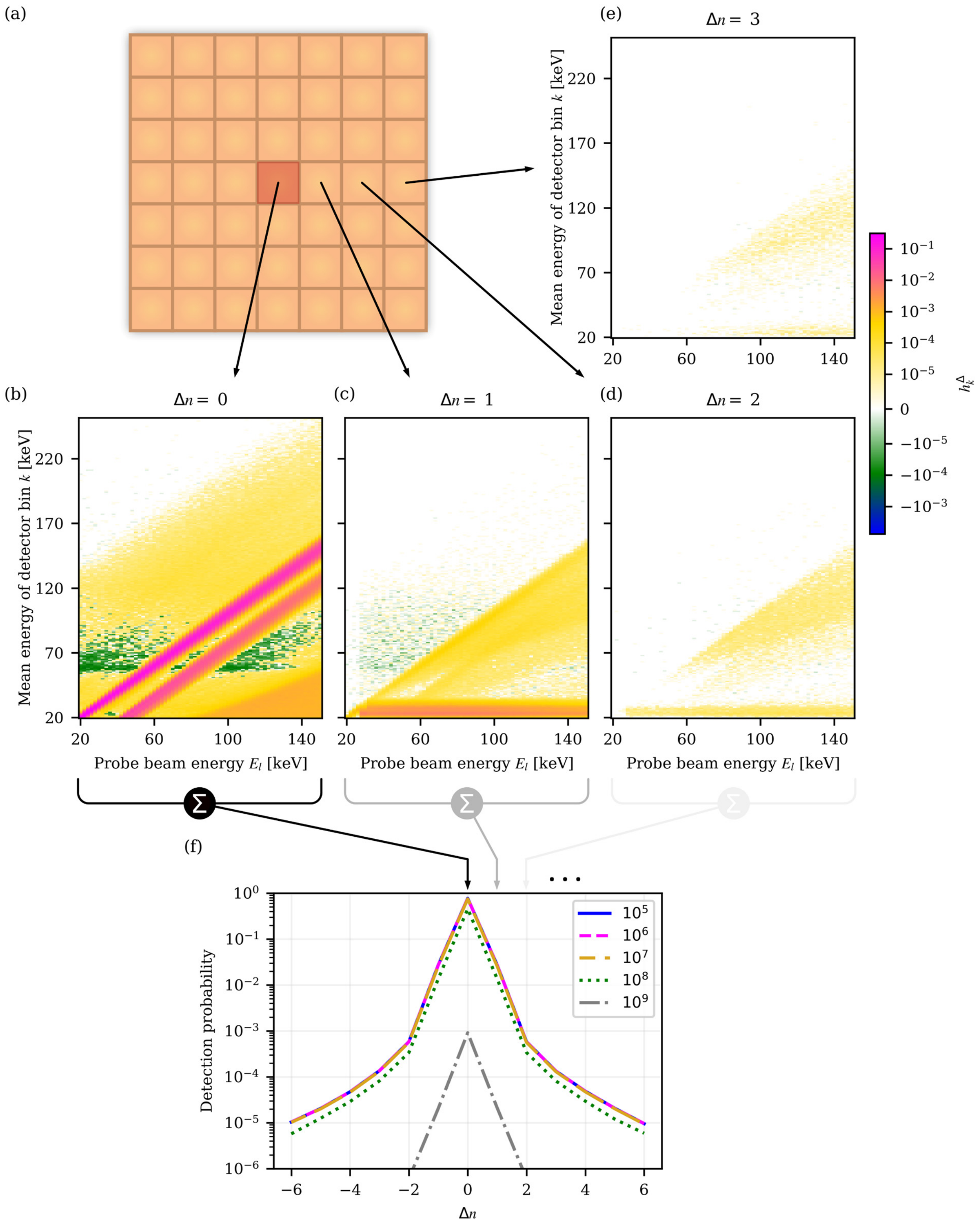

Figure S7: *Caption on next page*

Figure S7: $h_k^{\Delta}(\Delta\boldsymbol{n}, \dot{\boldsymbol{\Phi}}_{\text{op}}, E_l)$, that is, the pPSF h_k^{Δ} for all registering energy bins k and all simulated probe beam energies E_l of the idealized direct-conversion detector, for the center pixel ($\Delta\boldsymbol{n} = 0$, (b)) and three neighboring pixels (c – e) as indicated in the schematic (a). Here, a paralyzable counting behavior and a total fluence rate of the operating spectrum $\dot{\boldsymbol{\Phi}}_{\text{op}}$ of 10^6 mm^{-2} s^{-1} is assumed. (e) shows the pPSF for the indicated fluence rates under the assumption that the incident probe beam has a white spectrum, i.e., all energies are equally likely, for a row of pixels (center pixel as well 6 neighboring pixels on both sides). (e) then gives the probability that a photon of the incoming probe beam is detected in any energy bin of the neighboring pixels. This is analogous to summing over $h_k^{\Delta}(\Delta\boldsymbol{n}, \dot{\boldsymbol{\Phi}}_{\text{op}}, E_l)$ for each pixel and normalizing by the number of energies in the probe beam spectrum.

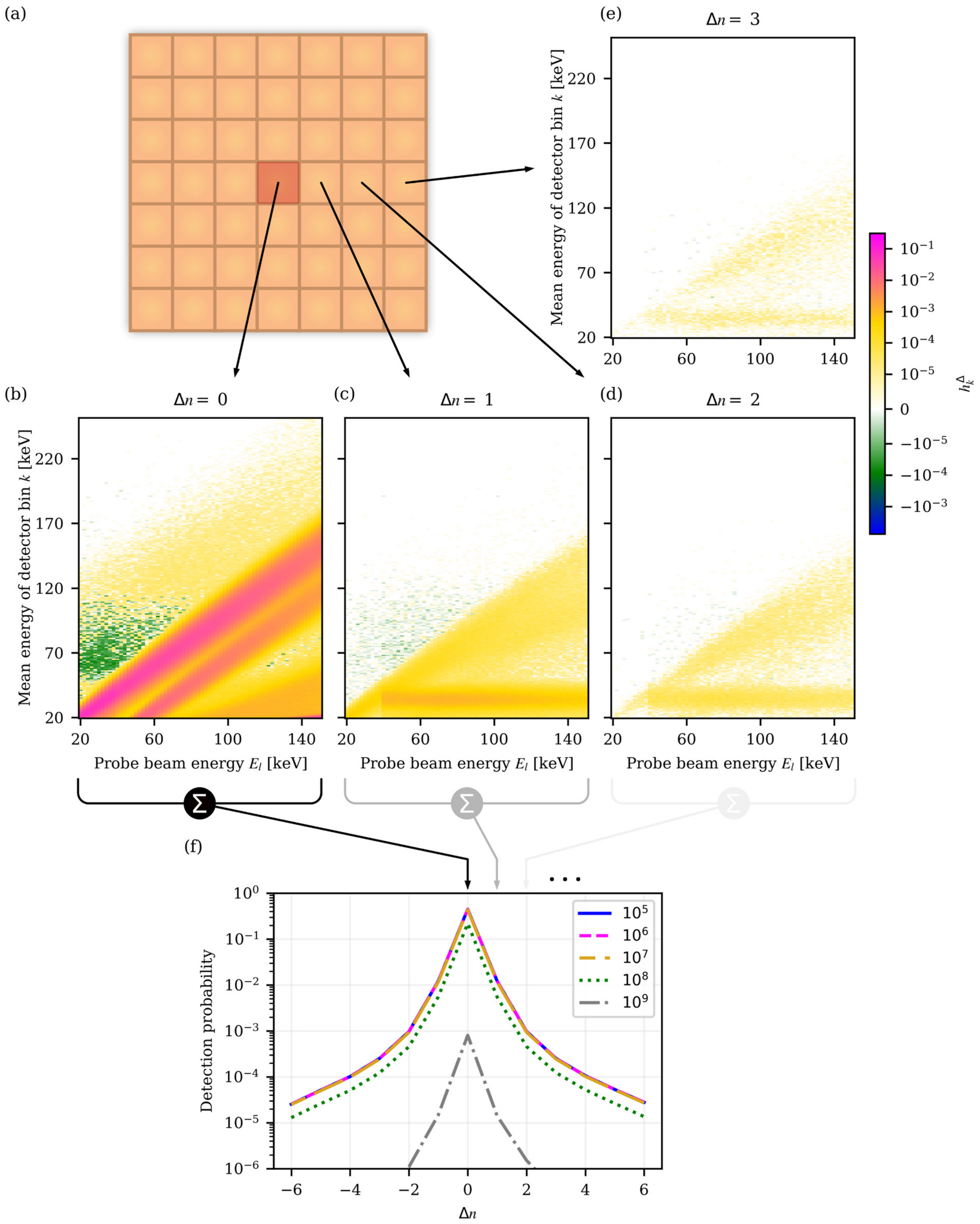

Figure S8: *Caption on next page*

Figure S8: $h_k^{\Delta}(\Delta\boldsymbol{n}, \dot{\boldsymbol{\Phi}}_{\text{op}}, E_l)$, that is, the pPSF h_k^{Δ} for all registering energy bins k and all simulated probe beam energies E_l of the idealized indirect-conversion detector, for the center pixel ($\Delta\boldsymbol{n} = 0$, (b)) and three neighboring pixels (c – e) as indicated in the schematic (a). Here, a paralyzable counting behavior and a total fluence rate of the operating spectrum $\dot{\boldsymbol{\Phi}}_{\text{op}}$ of 10^6 mm^{-2} s^{-1} is assumed. (e) shows the pPSF for the indicated fluence rates under the assumption that the incident probe beam has a white spectrum, i.e., all energies are equally likely, for a row of pixels (center pixel as well 6 neighboring pixels on both sides). (e) then gives the probability that a photon of the incoming probe beam is detected in any energy bin of the neighboring pixels. This is analogous to summing over $h_k^{\Delta}(\Delta\boldsymbol{n}, \dot{\boldsymbol{\Phi}}_{\text{op}}, E_l)$ for each pixel and normalizing by the number of energies in the probe beam spectrum.

S2.2 CNR for realistic energy bin widths

While Figure 8 (Figure 9) shows the contrast (CNR) assuming energy bins with widths of 1 keV, Table S1 (Table S2) lists the contrast (CNR) obtained when a more realistic energy binning is applied (see Equation (3)). It compares the results for a low energy bin of [20;65) keV (including lower boundary, excluding upper boundary), and two high energy bins of either [65; 120) keV, that is, including events up to the highest photon energy emitted by the source, or [65; 250] keV, that is, including also pile-up events.

For the contrast, for an energy bin of [20;65) keV the value of the contrast first drops in magnitude and then switches sign if the fluence rate of the operating spectrum is increased, whereas for energy bins of [65; 120) keV and [65; 250) keV the contrast first increases in magnitude due to the influence of pile-up and then switches sign.

For the CNR, for energy bins of [20;65) keV and [65; 120) keV the value of the CNR first drops in magnitude and then switches sign if the fluence rate of the operating spectrum is increased, whereas for an energy bin of [65; 250) keV the CNR first increases in magnitude due to the influence of pile-up and then switches sign.

Table S1: Contrast C_k due to the insertion of a small water/iodine lesion for realistic energy bins for both the idealized direct-conversion detector (iDCD) and idealized indirect-conversion detector (iICD). The table shows the results for three realistic energy bins with the given intervals, and for incident total fluence rates of the operating spectrum $\dot{\mathbf{\Phi}}_{\text{op}}$ between 10^6 – 10^9 mm^{-2} s^{-1}. For both detectors, a non-paralyzable-like behavior was assumed. The lesion is assumed to be characterized by $\rho \cdot x = 0.0007$ g/cm^2.

Fluence rate [mm^{-2} s^{-1}]	Energy bin intervals [keV]					
	iDCD			iICD		
	[20; 65)	[65; 120)	[65; 250)	[20; 65)	[65; 120)	[65; 250)
10^6	−0.050	−0.029	−0.109	−0.039	−0.029	−0.081
10^7	−0.048	−0.031	−0.215	−0.039	−0.030	−0.160
10^8	−0.024	−0.027	−0.227	−0.017	−0.026	−0.240
10^9	0.093	0.198	0.474	0.029	0.108	0.379

Table S2: Contrast-to-noise ratio $C_k/\sigma(C_k)$ due to the insertion of a small water/iodine lesion for realistic energy bins for both the idealized direct-conversion detector (iDCD) and idealized indirect-conversion detector (iICD). The table shows the results for three realistic energy bins with the given intervals, and for incident total fluence rates of the operating spectrum $\dot{\mathbf{\Phi}}_{\text{op}}$ between 10^6 – 10^9 mm^{-2} s^{-1}. For both detectors, a non-paralyzable-like behavior was assumed. The lesion is assumed to be characterized by $\rho \cdot x = 0.0007$ g/cm^2.

Fluence rate [mm^{-2} s^{-1}]	Energy bin intervals [keV]					
	iDCD			iICD		
	[20; 65)	[65; 120)	[65; 250)	[20; 65)	[65; 120)	[65; 250)
10^6	−73	−84	−90	−61	−62	−68
10^7	−72	−83	−101	−60	−61	−73
10^8	−35	−74	−128	−39	−51	−85
10^9	3.7	7.8	47	4.4	6.3	31
